# Tuning of the Electro-Optical Properties of Tetraphenylcyclopentadienone via Substitution of Oxygen with Sterically-Hindered Electron Withdrawing Groups

**DOI:** 10.1038/s41598-019-49303-w

**Published:** 2019-09-04

**Authors:** Carmine Coluccini, Puliparambil Thilakan Anusha, Hsin-Yi Tiffany Chen, Sheng-Lun Liao, Ying Kuan Ko, Atsushi Yabushita, Chih Wei Luo, Yoke Mooi Ng, Yit Lung Khung

**Affiliations:** 1 Institute of New Drug Development, China Medical University, No.91 Hsueh-Shih Road, Taichung, 40402 Taiwan; 2 Department of Electrophysics, National Chiao Tung University, 1001 University Road, Hsinchu, 30010 Taiwan; 3 Department of Physics, School of Advanced Sciences, Vellore Institute of Technology, Vellore, 632 014 India; 4 Department of Engineering and System Science, National Tsing Hua University, Hsinchu, 30013 Taiwan; 5 Molecular Science Center, GGA Corp, Taipei, 11493 Taiwan; 6 Taiwan Consortium of Emergent Crystalline Materials (TCECM), Ministry of Science and Technology, Taipei, Taiwan; 7 Department of Biological Science and Technology, China Medical University, No.91 Hsueh-Shih Road, Taichung, 40402 Taiwan

**Keywords:** Analytical chemistry, Coordination chemistry, Organic chemistry, Photochemistry, Supramolecular chemistry

## Abstract

In this report, the substitution of the oxygen group (=O) of Tetraphenylcyclopentadienone with =CR_2_ group (R = methyl ester or nitrile) was found to have tuned the electro-optical properties of the molecule. Although both groups are electrons withdrawing in nature, their absorption from UV-vis spectra analysis was observed to have been blue-shifted by methyl ester substitution and red-shifted by nitrile substitution. Interestingly, these substitutions helped to enhance the overall intensity of emission, especially in the context of methyl ester substitution whereby the emission was significantly boosted at higher concentrations due to hypothesized restrictions of intramolecular motions. These observations were explained through detailed descriptions of the electron withdrawing capability and steric properties of the substituents on the basis of density functional theory calculations.

## Introduction

Small or medium-sized organic molecules with an extended π-conjugation had been studied as photonic materials for light-emitting diodes, bioanalysis, thin film transistors, and photovoltaic devices^1–13^. This was due to high electron mobility empowering these molecules with ‘metallic-like’ characteristics as well as a highly accessible HOMO-LUMO energy gap that enabled absorption of UV-visible light. The overall shape and the dynamics of the π-conjugated skeleton arising from electron-withdrawing or donor functional groups is highly crucial in the determining electro-optical properties of the materials. This in turn also suggested that well designed organic synthesis can fine tune electro-optical properties. Furthermore, organic compounds are highly soluble and thus be easily applied for fabricating devices. Consequently, it is this unique ability to customize organic conjugated molecules as potential replacement of the inorganic semiconductors that provided the necessary impetus for detailed studies into π-conjugation systems^14^.

Conventional π-conjugated compounds are essentially planar D-π-A molecules (D for electron donating group and A for electron accepting group) where the excited state can exhibit charge separation. In recent years, a major breakthrough in the field of organic emitters was the synthesis and functionalization of the helical shaped Hexa-phenylsilole (HPS) and Tetraphenyl-ethylene (TPE) that are both π-conjugated molecules but not of the nominal D-π-A^15–23^. Interestingly, these compounds have turned out to possess many practical properties. Unlike traditional D-π-A emitters, they have exhibited an increased level of emission under higher concentration and condensed phase. This phenomenon are often described through mechanisms involving restricted intramolecular motions. Consequently, D-π-A molecules without exhibiting any planar structures are widely investigated due to this increased emission in concentrated solutions^24–27^, and such increment are often rationalized through the formation of J-aggregates that in turn increases and redshift the emission^28^.

**Figure 1:**
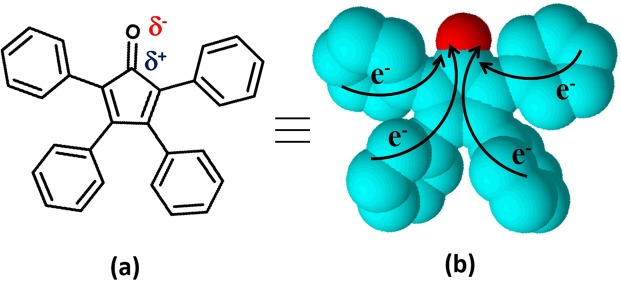
Molecular structure with polarity (**a**) and three-dimensional helical shaped conformation (**b**) with the electron mobility (e-) of the Tetraphenylcyclopentadienone.

In photovoltaics, helically shaped compounds have been frequently examined as effective replacement for fullerene in Bulk Heterojunction Solar Cells^29^. The helical shape allows for smaller domain sizes while still providing sufficient electron mobility at the same time. Moreover, these compounds are typically highly soluble in organic solvents even without long aliphatic chains, which is very useful during the device fabrication process. Some of the advantages of these molecules are the similarity in properties akin to that of fullerenes with smaller molecular spatial volumes as well as the reduced costs in terms of production and purification. Tetraphenylcyclopentadienone is one such helical shaped π-conjugated molecule that features unidirectional electron mobility due to the presence of the polar -C=O group (Fig. 1). Its range of UV-visible light absorption falls within the region 250–600 nm that is broader compared with other π-conjugated full organic molecules. As shown in Fig. 1, the molecular structure comprises of a cyclopentadienone linked with four phenyl groups.

The electronic properties of the Tetraphenylcyclopentadienone and its derivatives obtained from the deliberate modification on the phenyl rings were previously reported^30–34^. These derivatives are subsequently utilized as synthetic intermediates for the production of polyphenylene dendrimers as well as building blocks for gelators that absorb and emit within the UV-visible frequencies^35–43^. To provide further insight toward the synthesis of new helical shaped π-conjugated compounds for electrooptical applications, herein, the effects from the substitution of the oxygen group with =CR_2_ moeities (where R is –COOMe and –CN) were investigated. The compounds examined in this study were 2,3,4,5-tetraphenylfulvenes where the carbon at position 6 is bonded to two electron withdrawing groups. Pentafulvene is a molecular module that through chemical modification of the carbon at position 6 would switch from aromatic to antiaromatic electronic configuration with substitution at pentene ring, thus enabling for the fine-tuning of the HOMO-LUMO gap^44–48^. We have shown that the conversion of tetraphenylcyclopentadienone to tetraphenyl pentafulvene with different substituents at the carbon 6 position can fine-tune electro-optical properties in wide range. The absorption and emission spectra, in conjunction with density functional theory (DFT) calculations and femtosecond pump-probe measurements, were corroborated to produce an overall picture regarding to the electronic distribution and the interaction of these compounds with light.

## Results and Discussion

### Synthesis of compounds 1a and 1b

**Scheme 1:**
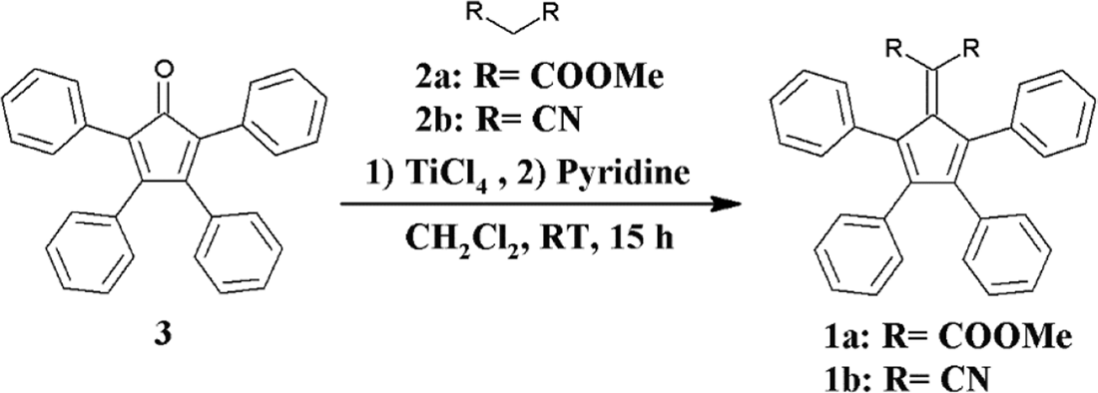
Synthesis of emitting molecules **1a** and **1b**.

The synthesis of the compounds **1a** and **1b** was as shown in Scheme 1. The treatment of Tetraphenylcyclopentadienone **3** and compound **2a** (Dimethyl malonate) or **2b** (Malononitrile) with TiCl_4_ and Pyridine allowed for the recovery of compounds **1a** or **1b** respectively. The compound **1b** is known in literature and two methods had been reported for the preparation. The procedure adopted in this work is based on the report by Andrew *et al*. and has been also utilized for the synthesis of **1a** with high yields obtained^49–51^.

### NMR Characterization

**Figure 2:**
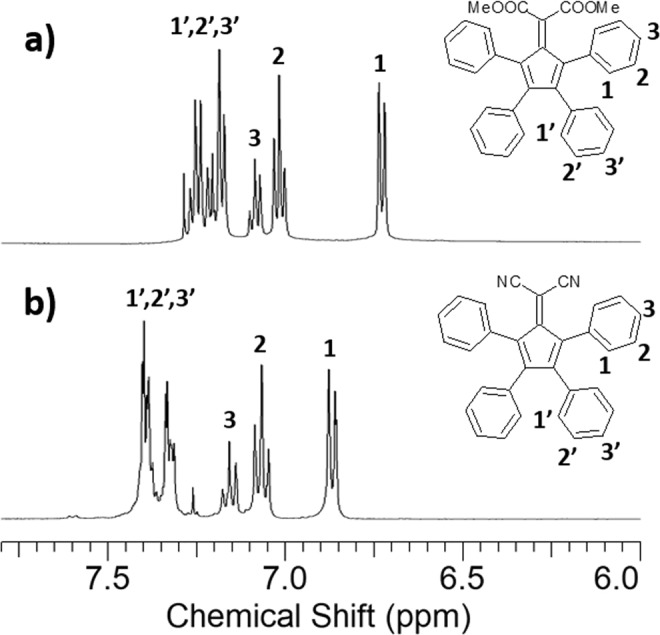
Aromatic 1HNMR signals of compounds (**a**) **1a** and (**b**) **1b**.

The aromatic 1H NMR signals of the compound **1a** were shifted at lower ppm to the respect **1b** (the protons 1, 2, 3 of **1a** are at 6.73, 7.02, 7.09 ppm, the protons 1, 2, 3 of **1b** are at 6.87, 7.07, 7.16 ppm, as displayed in Fig. 2) due to the highest electron-withdrawing character of the -CN group compared to -COOMe, as deduced from the values of the Hammet substituent constants σ_p_ and σ_m_ of the two functional groups (σ_m_ and σ_p_ are 0.56 and 0.66 for –CN, 0.37 and 0.45 for –COOMe)^52^. The proton signals of the phenyl rings closer to the electron-withdrawing groups were distinguishable (protons 1, 2, 3 in Fig. 2). The signals of the other phenyl groups were found to have overlapped (protons 1′, 2′, 3′ in Fig. 2). In compound **3**, the 1H NMR signals of protons 1, 2, 3, as reported previously, were observed to exhibit highest chemical shifts with respect **1a** and **1b** (the protons 1 are at 6.90–6.94 ppm, the protons 2 are at 7.15–7.19 ppm and the protons 3 are at 7.23–7.26 ppm together with the protons 1′, 2′ and 3′)^53^.

### Optical property analysis of compounds 1a, 1b and 3

#### Absorption profile

**Figure 3:**
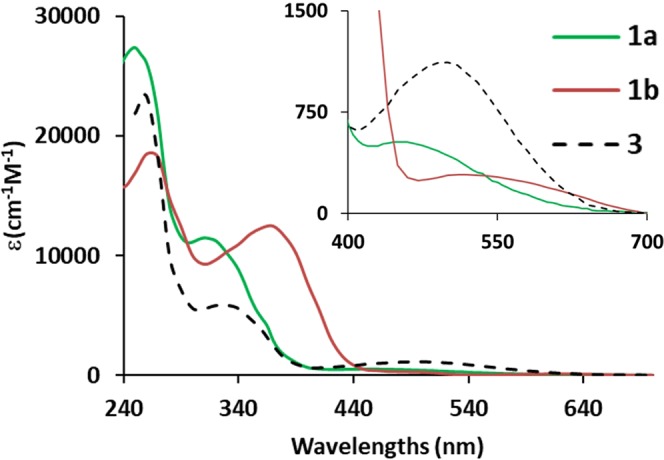
UV-vis spectra of **1a**, **1b**, **3** in CH_3_CN, specral range of 240–700 nm and in the inset for spectral range of 400–700 nm.

The UV spectra of **1a**, **1b** and **3** in CH_3_CN are as shown in Fig. 3 while the spectra taken in other solvents (Toluene, DMF and MeOH) are provided in Fig. S5. All absorption peaks as observed were binned into three separate regions: 250–260 nm, 320–380 nm, 500–510 nm and their λ_max_ were labeled as λ_1_, λ_2_, λ_3_ respectively (Table 1). The λ_1_ was not detectable in all solvent due to the cut-off point of the solvent. Compound **3** exhibited signals at 330–340 nm (λ_2_) and 500–510 nm (λ_3_) while the variation was highly dependent on the solvent type. Dependence on compounds was found to be as follows. Compound **3**’s λ_3_ was clearly discernible in all solvents although it displayed a low molar absorption coefficient compared to the other compounds’ signals at λ_2_. The absorption spectrum of **1a** at 320–330 nm (λ_2_) and 460 nm (λ_3_) were observed at shorter wavelengths with respect to compound **3**. **1b** exhibited absorption bands at longer wavelengths compared to the other two compounds at 370–380 nm (λ_2_) and 500–545 nm (λ_3_). The molar absorption coefficient at λ_3_ for both compounds **1a** and **1b** has been lower than that at λ_2_ with partially overlapping signals in Toluene, CH_3_CN and CH_2_Cl_2_. On the other hand, in polar solvents such as DMF and MeOH, the peak was observed to be broadened and it was not easily discernable. The molar absorption constants at λ_2_ have been highest for **1a** and **1b** while highest for **3** at λ_3_.

**Table 1:** Optical properties of the compounds 1a, 1b and 3.

Solvent (dipolar moment)	1a		1b		3	
	λ_abs_^[a]^ nm λ_1_, λ_2_, λ_3_	λ_em_^[b]^ nm (Φ^[[c]^)	λ_abs_^[a]^ λ_1_, λ_2_, λ_3_	λ_em_^[b]^ nm (Φ^[c]^)	λ_abs_^[a]^ λ_1_, λ_2_, λ_3_	λ_em_^[b]^ nm (Φ^[c]^)
Toluene (μ = 0.4 D Δ*f* = 0.015)	330 (13) 460 (0.8)	435 (0.13)	380 (15) 500 (0.4)	435 (0.34) 500 (0.4)	330 (7.5) 510 (1.5)	435 (0.0031)
CH_2_Cl_2_ (μ = 1.8 D Δ*f* = 0,218)	250 (19) 320 (8.0) 460 (0.03)	435 (0.13)	270 (17) 380 (13) 545 (0.03)	435 (0.045)	260 (26) 330 (6.3) 510 (1.1)	435 (0.015)
DMF (μ = 3.8 D Δ*f* = 0,274)	320 (13) 460 (0.02)	—	375 (15) 500 (0.02)	—	330 (7.5) 510 (1.3)	—
CH_3_CN (μ = 3.2 D Δ*f* = 0,305)	250 (27) 320 (12) 460 (0.5)	430 (0.0095)	260 (20) 370 (14) 500 (0.3)	430 (0.061)	260 (23) 330 (5.2) 500 (0.5)	435 (0.001)
MeOH (μ = 1.7 D Δ*f* = 0,309)	322 (16) 464 (0.05)	430 (0.01)	374 (19) 500 (0.05)	435 (0.02)	332 (7.3) 502 (1.7)	435 (0.001)

^[a]^Average values of absorption wavelengths (nm) at different concentrations. The intensities are reported in brackets, the units are M^−1^cm^−1^ × 10^3^. In Toluene and DMF, the λ_1_ is not visible for the cut off of the solvents. ^[b]^Emission generated from excitation at the λ_2_ of absorption and concentrations ~2–5 × 10^−6^ M. ^[c]^Quantum yield measured at concentrations ~5 × 10^−6^ M, as reference Phenothiazine was used which value of Φ is 0.01^56^.

#### Emission spectra

The excitation wavelength of fluorescence was set at λ_2_ for all solvents types and all compounds. All compounds of **1a**, **1b** and **3** had exhibited fluorescence at 430–435 nm in Toluene, CH_3_CN, MeOH, CH_2_Cl_2_. Compound **3** displayed the weakest fluorescence signal among all three compounds for all solvents. Interestingly, in DMF (the solvent with the highest dipolar moment), no fluorescence was observable for all compounds. The intensity of fluorescence of all compounds decreased with increasing polarity of the solvents. The quenching of fluorescence in DMF may be due to the high dipolar moment of the solvent as well as other reasons as previously reported in the literature, i.e. the interactions of the energetically excited molecules of emitter with the molecules of solvent or the presence of impurities in the solvent (even in the commercially available high purity DMF) that could result in the quenching of emission of the solute^54^. Table 1 listed all the respective optical properties of the compounds **1a**, **1b**, and **3**. The values of the dipolar moments (μ) and orientational polarizability (Δ*f*) of the solvents are as reported from literature^55^.

**Figure 4:**
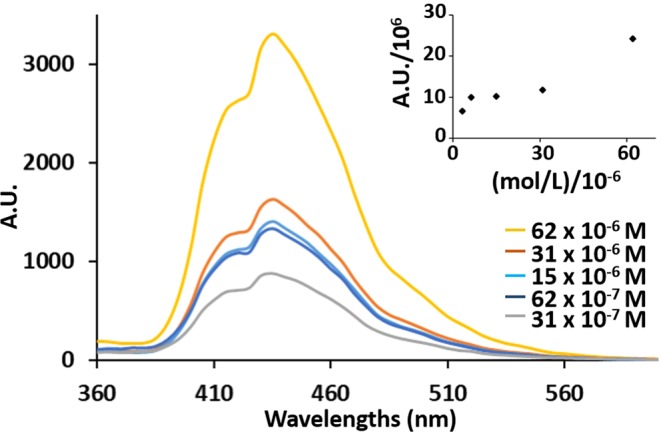
Fluorescence spectra of **1a** in Toluene at different concentrations, the inset is the plot of Fluorescence intensities (as area under the curve) of **1a** in Toluene vs. concentration.

All emission spectra of the compounds, in different solvents, and at different concentrations are shown in the Fig. S6 of SI. Compound **3** exhibited fluorescence enhancement with concentration in Toluene and CH_2_Cl_2_, while in MeOH and CH_3_N the discerning of the precise peak maxima is technically challenging because of low fluorescence intensity The compound **1a** exhibits highest fluorescence intensity when the concentration increased from 30–60 × 10^−7^ M to 30–60 × 10^−6^ M in all solvents with CH_2_Cl_2_ being the exception. The fluorescence spectra at six different concentrations of the compound **1a** in Toluene is as displayed in Fig. 4. There was a direct correlation between intensity of the fluorescence and the concentration which was more intense at highest concentration. The plot of the fluorescence maxima at different concentration shown as inset of Fig. 4, could be fitted with a Hill equation. It was possible to examine the emission of compound **1a** in Toluene with the same modality of other phenomena where physical measurement depends on the supramolecular aggregation between molecules^25,57,58^. The intermolecular aggregation increased the intensity of emission and the Eq. (1) can be used to correlate with the observed graph as shown in Fig. 4b:

1 $$\Delta Em=\Delta E{m}_{max}\,\frac{{[x]}^{n}\,{K}_{a}}{(1+{K}_{a}{[x]}^{n})}$$

Where *ΔEm* is the variation of the intensity of emission, [*x*] is the concentration of the free molecule in solution, ${K}_{a}$is the value of the constant of formation of aggregate. The Hill coefficient for the curve of Fig. 4b is *n* = 0.85. The value *n* < 1 indicates not-cooperative interaction and this suggested that the aggregates are oligomers. Compound **1b** was found to demonstrate the quenching of the fluorescence when the concentration was increased in all solvents, with CH_2_Cl_2_ being the exception.

### Femtosecond time resolved spectroscopy analysis

In order to study the dynamics under photoexcitation and to gain better insights into the mechanism of emission of compounds **1a** and **1b**, the degenerate femtosecond pump-probe measurements^59^ were performed in DMF and Toluene. The solutions were taken in a quartz cuvette of 1mm thickness. The sample solutions were excited using the pump pulse (the spectral and temporal profile are given in Fig. S8) and difference in the probe transmittance (ΔΤ) with and without the presence of pump, were collected at each delays of pump with respect to probe pulse. The differential absorbance were calculated using the equation: ΔA = − log (1 + ΔT/T) where T is the sample transmittance in the absence of pump. The differential absorbance (ΔA) for selected probe wavelengths were plotted at each delays and time constants were extracted by fitting the data using exponential functions.

**Figure 5:**
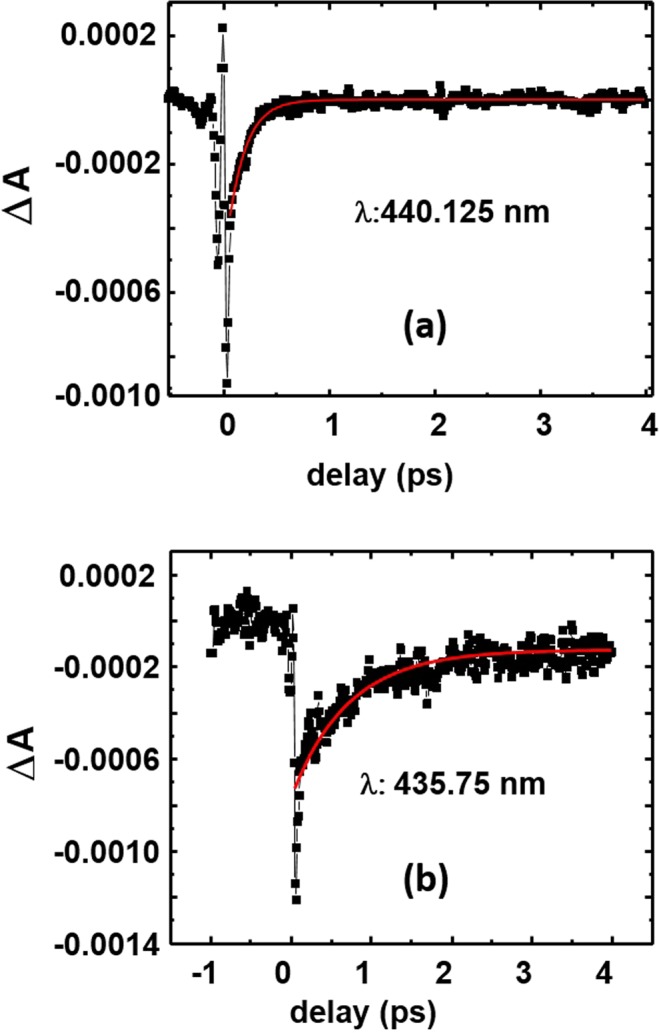
The temporal dynamics of (**a**) compound **1a** at probe wavelength of 440 nm and (**b**) compound **1b** at probe wavelength of 435 nm in DMF solutions. The concentration of the solutions was 0.5 mM. The red line is the exponential fitting of the decay.

In DMF, there was a strong negative signal (ΔA < 0) in the UV range (<450 nm). Since this wavelength range overlap with resonance absorption of the respective compounds, the signal could be assigned to a ground state bleaching^60^. The temporal dynamics at probe wavelength of ~440 nm for compounds **1a** and **1b** in DMF are displayed in Fig. 5. The recovery lifetimes of the ground state population were estimated by the fitted time constants. The time constants were found to be in the range of 0.16–0.28 ps for **1a** and 0.60–1.08 ps for **1b** in DMF solvent. In DMF, both compounds had shown no photoluminescence and the excited population possibly decayed non-radiatively.

**Figure 6:**
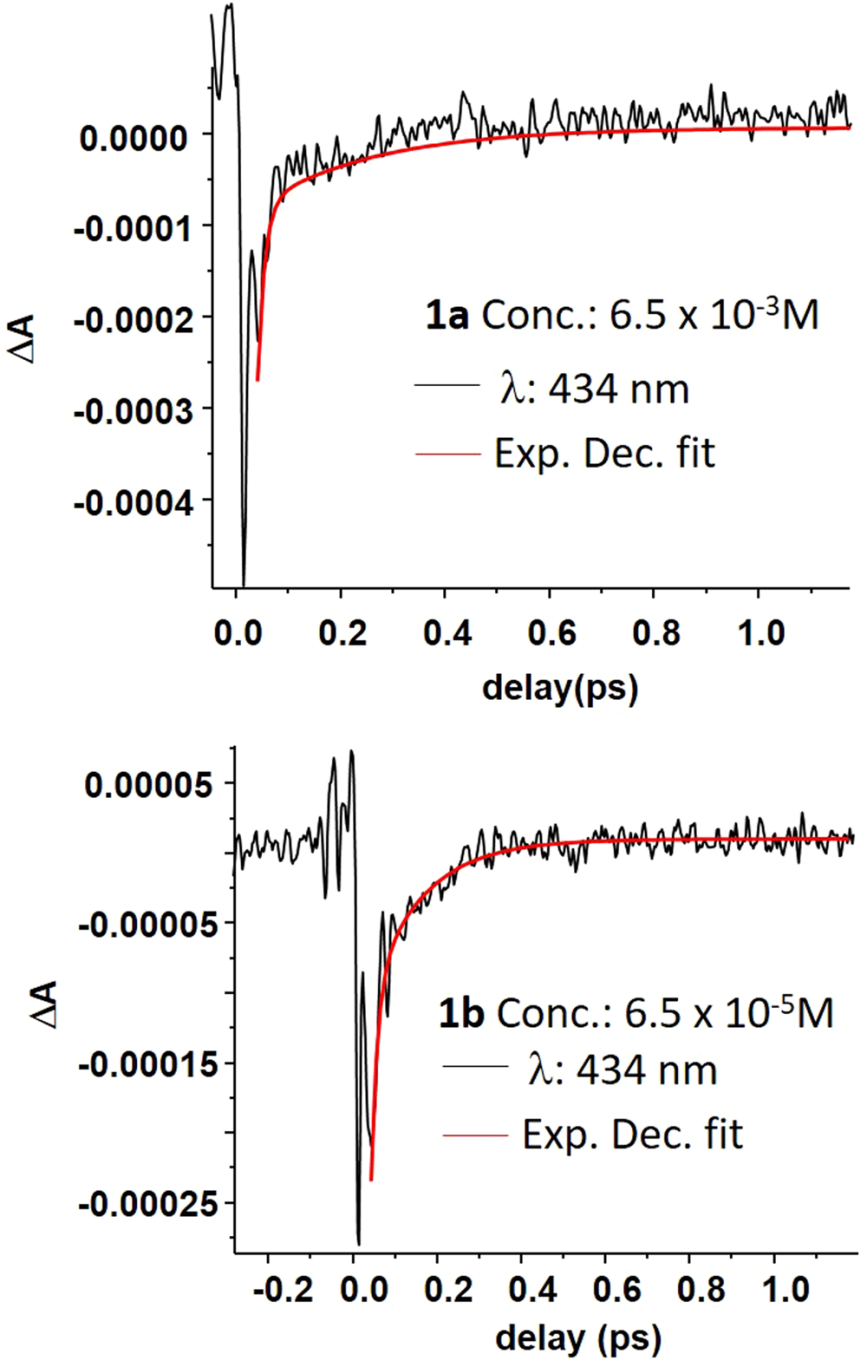
The temporal dynamics of compound **1a** and compound **1b** in Toluene at a probe wavelength of 434 nm.

**Figure 7:**
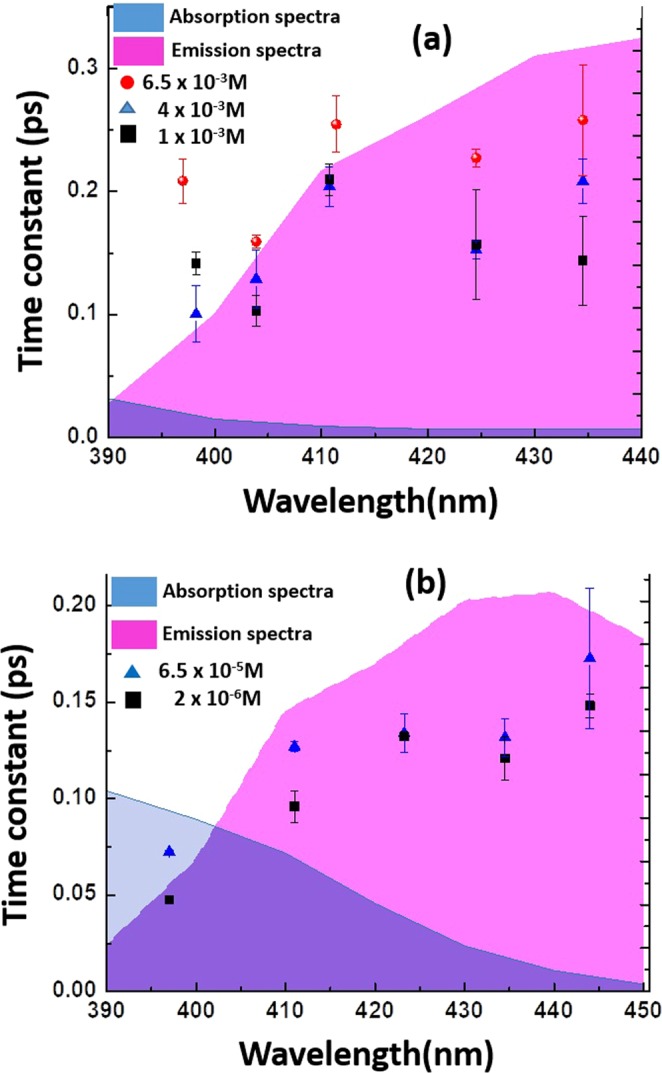
The spectral dependent time constants from pump probe measurements for different concentrations of compounds (**a**) **1a** and (**b**) **1b** in Toluene. The pink shaded region show the emission band and the blue shaded region show the absorption of the respective compounds in Toluene.

On the other hand, the temporal dynamics in the range of 380–460 nm in Toluene for both compounds (**1a** and **1b**) had shown existence of much faster decay lifetime (0.07–0.25 ps) which was not found in DMF. The negative signal (ΔA < 0) in the region 380–460 nm for compound **1a** and 425–460 nm for compound **1b** could be assigned to the stimulated emission from the excited state. However, there is an overlap of absorption band leads to a ground state bleaching in the pump probe signal (ΔA) at lower wavelengths. The temporal dynamics of the compounds **1a** and **1b** in Toluene at probe wavelength of 434 nm were displayed in Fig. 6. The decay was fitted by a bi-exponential function giving two time constants. The first time constant was comparable with the pulse duration (~ 8.1 fs as shown in Fig. S8). The second time constants were assigned to the decay of excited state population. These decay time constants were found to be 0.1–0.2 ps for **1a** and 0.07–0.17 ps for **1b** in Toluene as shown in Fig. 7. However in the lower wavelength region (<410 nm for **1a** and <430 nm for **1b**), due to the overlap of resonance absorption, there would be a contribution of ground state bleach signal in addition to the stimulated emission and hence the time constants from the aforementioned region can not be considered to discuss about the excited state population recovery. The pump probe measurements were, further carried out and analyzed at different concentrations to observe the effect of aggregation to the decay time constants in Toluene solvent.

The time constants at selected probe wavelength for three different concentrations for Toluene solution of compound **1a** (6.5 × 10^−3^ M, 4 × 10^−3^ M and 1 × 10^−3^ M) and **1b** (3.2 × 10^−3^ M, 6.5 × 10^−5^ M, 2 × 10^−6^ M) were displayed in Fig. 7a,b respectively. It was observed that when the concentration of **1a** was increased, the time constants were also increasing gradually at the probe wave lengths which were overlapping with the emission band (420–440 nm) of **1a**. However, for compound **1b**, the time constants remained almost constant and with the highest concentration (3.2 × 10^−3^ M) of **1b** we were unable to observe any pump-probe signal as the quenching of emission might have occurred at this concentration. The observations showed that the radiative decay takes longer time in the aggregation phase (i.e at higher concentration in Toluene) of **1a** through RIM (restricted intramolecular motions) process and hence the enhancement of emission due to the longer time of the excited state.

### Fluorescence analysis in solvents with different viscosity

**Figure 8:**
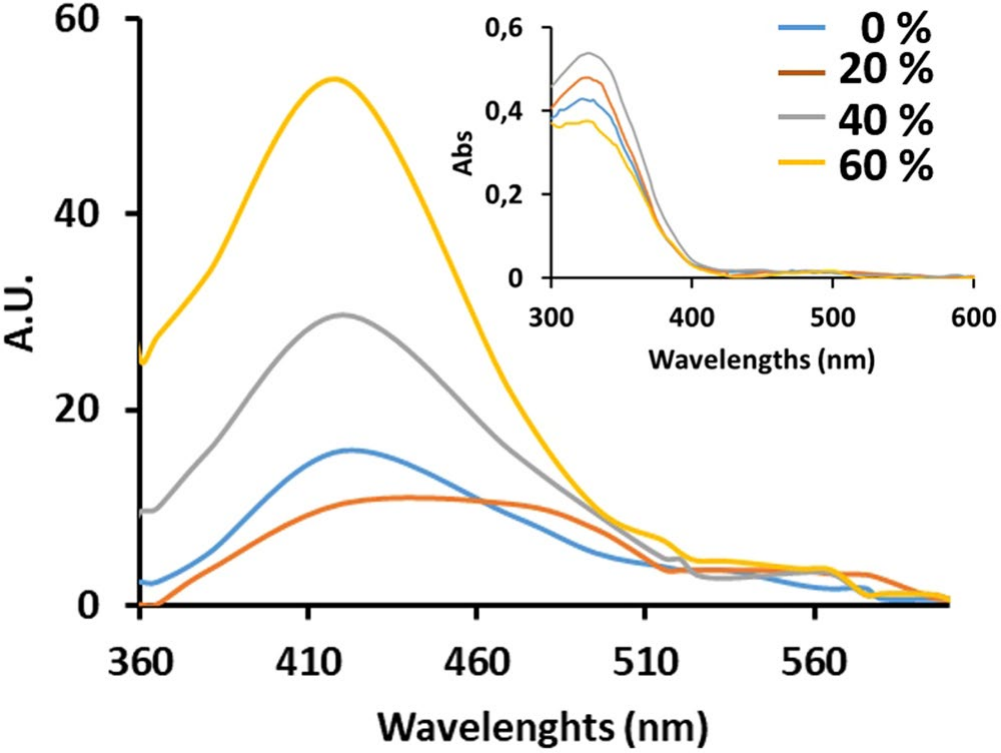
PL spectra and UV-vis spectra (inset figure) of **1a** 30 × 10^−7^ M in mixtures with different rates of MeOH/Glycerol. The increasing percentage of Glycerol from 0% to 60% with the consequent increase of solution viscosity does not produce an enhancement of UV-vis absorption but an increase of emission when the percentage of Glycerol is 40–60%.

The fluorescence of the compounds **1a** and **1b** has been analyzed in solutions at different viscosity, as showed in Fig. 8, in order to study the emission in environments with different rigidity. It is expected that the increased viscosity of solutions (increased rigidity of environment), restrict the intramolecular motions. A diluted solution of **1a** in mixtures Methanol/Glycerol has resulted in more emission when the viscosity was increased with higher the glycerol percentage. The increasing of emission is not detected when the percentage of glycerol has been increased from 0 to 20%, but increasing at 40% and 60% enhanced the emission. The solutions of **1b** (Fig. S15) display a light increasing of emission when the percentage of glycerol was 40% and 60% but the effect is less clear respect compound **1a**. The experiments show that in compound **1a**, the RIM increases the emission.

### Electronic structure analysis

#### Frontier molecular orbitals

**Figure 9:**
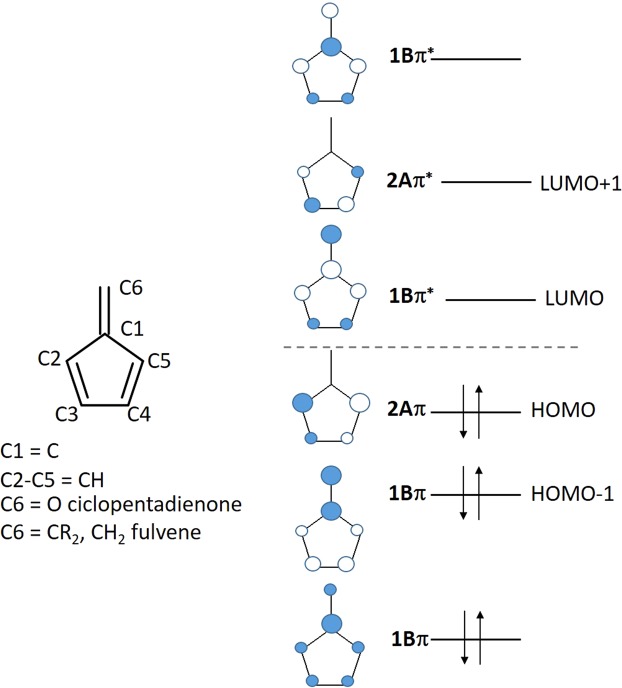
Schematic representation of the Molecular Orbitals of fulvene and cyclopentadienone as reported in the literature^60^.

In this computational section, we aim to qualitatively gain insight into the correlation between the substitution effects on molecular structure and maximum wavelength observed in adsorption spectrum through computing their geometry properties, the shapes and the energies of the frontier molecular orbitals (MOs) among compounds **1a**, **1b** and **3**. Based on Potters’s and Hughes’s study on the good correlation of the DFT calculated HOMO-LUMO gap with the experimental absorptions of compound **3**^33^, instead of using the time-dependent (TD) DFT approach, we connected the gaps of HOMO/LUMO, HOMO/LUMO+1, HOMO-1/LUMO with the major contribution of the peaks near visible region; such a descriptor with lower computational cost will be beneficial to the future molecular design for optical-electron properties. In this study, the MOs of the compounds **1a**, **1b** and **3** can be considered as perturbations of the fulvene core (**1a** and **1b**) and cyclopentadienone core (**3**) due to replacing hydrogens with phenyl rings at positions C2, C3, C4, and C5 in Fig. 9 and replacing =CH_2_ with =CR_2_ and =O at position 1. Before analyzing the MOs of the compounds with phenyl rings, we firstly describe the MOs of fulvene as previously reported by R. E. Duke’s group and was schematically represented in Fig. 9 ^61^. In Fig. 9, the six MOs of the fulvene are provided by the combination of the five p orbitals of the cyclopentene carbons and one p orbital of the exocyclic carbon; in the case of the cyclopentadienone, the exocyclic p orbital is provided by the oxygen. The six π-electrons of the fulvene occupy the three highest occupiedMOs.

**Figure 10:**
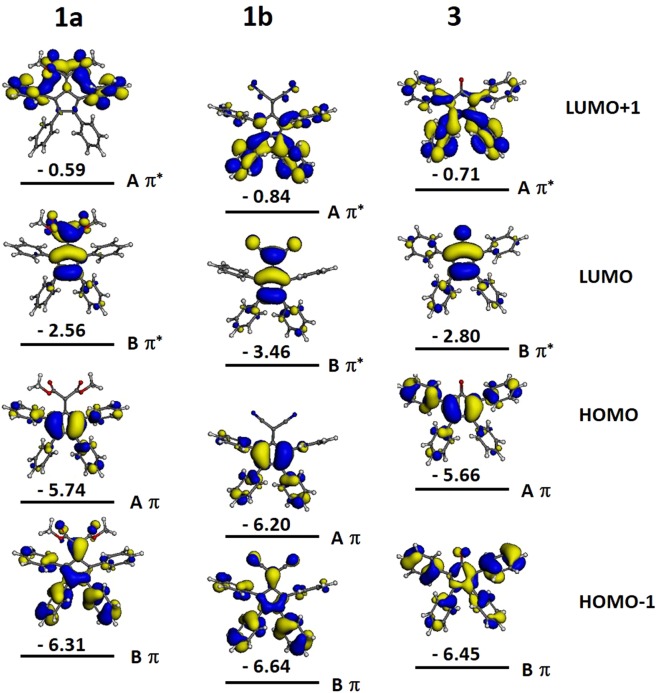
Graphic representation of the MO calculation results for the frontier MOs of compounds **1a**, **1b** and **3**. The values refer to their energy levels in eV.

The MOs of compounds **1a**, **1b** and **3** and their energies are represented in Fig. 10. The strongest perturbation of the phenyl rings on the orbitals of the fulvene is expected when the reciprocal disposition is coplanar.

The optimized geometries depicted in Fig. S16 show that the dihedral angle between the phenyl rings in position 2 and 5 of the cyclopentene ring and the fulvene group are 39° for compound **3**, 80° for compound **1b**, 63° for compound **1a**. The geometries can be explained with the steric hindrance of the groups in position 6. The size of the oxygen in compound **3** (=O) is smaller than the =CR_2_ groups of the other two compounds and, as consequence, the phenyl groups in position 2 and 5 of the cyclopentene ring are more free to adopt coplanar disposition respect to the fulvene. The =C(CN)_2_ group of compound **1b** exhibit stronger interaction with phenyl rings than the =C(COOMe)_2_ of **1a** because the –CN groups are coplanar with the cyclopentadiene and in the proximity of the phenyl groups in position 2 and 5. In compound **1a** the rotation of the –COOMe group place the –C=O and the –OMe out of the plane of the cyclopentadiene. The electron density of orbital HOMO Aπ is the highest on the carbons of cyclopentadiene and is the most destabilized by the perturbation of the phenyl rings. As a consequence, the HOMO Aπ orbitals of compound **3** and **1b** are respectively the most and the less destabilized. These outcomes demonstrate that importance of the steric hindrance of the R group in determining the electronic distribution along the molecular structure and as consequence in tuning the electro-optical properties of the compounds.

The electrons of the group that is of linked to the carbon 1 of the cyclopentadiene destabilize the orbital LUMO Bπ* which electron density is highest on position 6. The =C(COOMe)_2_ group in compound **1a** contain four oxygen atoms and eight electron lone pairs, =O of compound **3** contains two lone pairs, the =C(CN)_2_ of compound **1b** contains two nitrogen atoms and two electron lone pairs. As a consequence, the compound **1a** exhibits the highest electron density and the highest destabilization of the orbital LUMO Bπ*. The lowest destabilization in compound **1b** respect compound **3** is due to the higher extension of the group where the electron lone pairs are placed.

#### Correlation between calculated wavelength and observed absorption maxima

**Figure 11:**
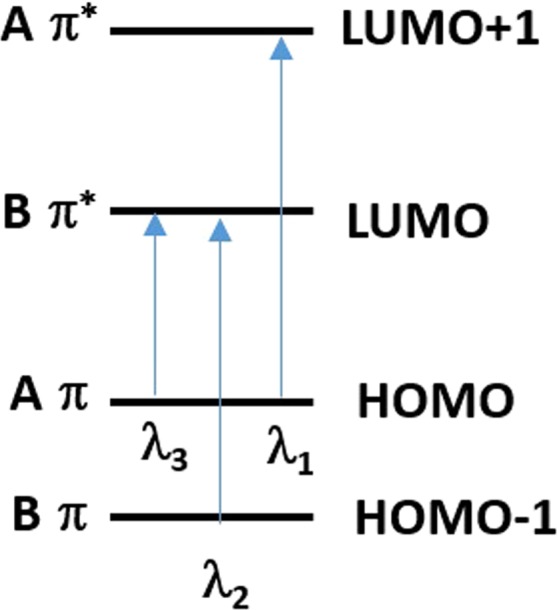
Correlation between calculated wavelengths and MOs transitions.

In this section, the correlation between calculated frontier MO energy differences and their corresponding wavelengths is illustrated in Fig. 11. The calculated λ_1_ (243 nm for **1a**, 232 nm for **1b**, 251 nm for **3**), λ_2_ (330 nm for **1a**, 389 nm for **1b** and 338 nm for **3**) and λ_3_ (391 nm for **1a**, 451 nm for **1b**, 433 nm for **3**) correlated well with the average absorption maxima in different solvents, λ_1_ (250 nm for **1a**, 260 nm for **1b**, 260 nm for **3**), λ_2_ (325 nm for **1a**, 375 nm for **1b**, 330 nm for **3**) and λ_3_ (460 nm for **1a**, 500 nm for **1b**, 505 nm for **3**) and are as shown in Table 2. A minor disparity between our calculated transitions HOMO Aπ - LUMO Bπ* with the observed experimental λ_3_ may be due to the effects of the solvent on the energy of the orbital HOMO Aπ that was not considered during our DFT calculations. The λ_3_ may have been influenced by the polarity of the solvent because the HOMO Aπ orbital is polar as previously described by Diedrich *et al*.^44^, and is stabilized by solvents with highest orientational polarizability and dipolar moment. In CH_3_CN the λ_3_ of compounds **1a**, **1b** and **3** were blue-shifted as compared to in Toluene and CH_2_Cl_2_ while broaden in DMF and MeOH. The calculated values of the transitions had also revealed that compound **1a**’s and **1b**’s λ_2_ and λ_3_ are partially overlapped (the calculated difference λ_3_-λ_2_ is only 60 nm) while in compound **3**, these are separated (the calculated difference λ_3_-λ_2_ is 100 nm) due to the strong destabilization of the HOMO Aπ that may have red-shifted the λ_3_.

**Table 2:** Computed values of the energy differences, expressed in nm, between the frontiers MOs of compounds 1a, 1b and 3.

Calculated Electronic transitions	1a	1b	3
LUMO + 1 Aπ* - HOMO Aπ (λ_1_ nm)	243	232	251
LUMO Bπ* - HOMO-1 Bπ (λ_2_ nm)	330	389	338
LUMO Bπ* - HOMO Aπ (λ_3_ nm)	391	451	433

In short, this consistent relationship between the three calculated wavelengths and absorption spectral peaks implies each of the three transitions identified associates with one predominant single electron excitation. Thus, it is reasonable to expect that there would be a correlation between the difference of corresponding molecular orbital energies by DFT and the observed transitions from experimental spectrum thought more complete contributions of MO to each excited state can be carried out by sophisticated TDDFT calculations^62^.

## Conclusions

In this work, we substituted the oxygen (=O) moiety of the cyclopentadienone with two different =CR_2_ groups bearing different steric hindrance. The substitution was shown to be an effective method for the tuning of the optical and the electronic properties of the molecule. Although the groups R of compounds **1a** and **1b** are both electrons withdrawing, the absorption peak shift with respect to compound **3** has been reversed between the two compounds (blue and a red-shifted accordingly). The compounds **1a** and **1b** have displayed an increased fluorescence when compared to the compound **3** due to having the most strained molecular arrangement. As confirmed with femtosecond time-resolved spectroscopy, the compound **1a** has exhibited increased fluorescence in more concentrated solutions. The fluorescence spectra of **1a** in solvent with different viscosity had revealed that the restricted intramolecular rotations may have contributed to the increase the fluorescence. The computational outcomes confirmed that the MOs states of –COOMe and –CN had also helped fine-tuning the electronic mobility along the molecular backbone due to the different steric interaction with the phenyl rings bonded to the carbons C2 and C5. In compound **3** the MOs of the cyclopentadienone are strongly extended on the phenyl rings that are bonded to the carbons C2 and C5. In compound **1a** the MOs of the fulvene unit are extended along the four phenyl rings and the =C(COOMe)_2_ group. In compound **1b**, the MOs of the fulvene core is extended on the =C(CN)_2_ group and on the phenyl rings bonded to the carbons C3 and C4. The straightforward DFT description allowed identifying the two excited states S_1_ (HOMO → LUMO) and S_2_ (HOMO-1 → LUMO) that have been found in other fulvene derivatives like diphenyl dibenzofulvene which absorption band lies in the wavelength range of 320–510 nm^63^. Fulvene unit displays a conical intersection seam between the fundamental state S_0_ and the excited state S_1_ that is accessed by twisting around the exocyclic bond C1-C6 of Fig. 9 ^64,65^. Blancafort’s group found that the emission of diphenyl dibenzofulvene depends on the accessibility to the conical intersection seam between the states S_1_ and S_0_^66,67^. In the solution, the conical intersection seam is more accessible than in solid state because the twisting trough the exocyclic bond C1-C6 is less hindered, and as consequence the emission is quenched more. Compounds **1a**, **1b** display that absorption and emission spectra are similar to diphenyl dibenzofulvene, indicating that the optical properties are determined by fulvene moiety. The lowest polarity (lowest solubilizing capability), the highest viscosity of solvent and the highest concentration of the solutions are the conditions that restrict the intramolecular motions and thus increase more clearly the emission of **1a** and less clearly the emission of **1b**. This evidence is coherent with the fact that the molecule **1b** is more hindered than **1a**. The intrinsic hindrance in **1b** (and restricted intramolecular conformational freedom) implies that the external conditions have lesser influence on intramolecular motions. The similarities with the diphenyl dibenzofulvene have demonstrated that the fluorescence depends on the conical intersection seam between the S_1_-S_0_ states and the C1-C6 twisting. The exact mechanisms could be analyzed with rigorous computational analyses that is not within the scope of this manuscript.

Herein, we demonstrated that substituted tetraphenylfulvenes can serve as valid starting point for the synthesis of novel D-π-A compounds and new emitting molecules. The compounds displayed tunable electronic properties in conjunction with easy manipulability that is conferred by non-coplanar disposition of the phenyl rings. These enhancements of emission by restricting of the intramolecular motion may also have an important role for biosensors, especially in terms of host-guest chemistry based docking sensors. A further work pertaining to this research that will be on the additional chemical modifications of the compounds **1a** and **1b** to serve as connections of the phenyl rings with electron-withdrawing or electron donor groups.

## Experimental Section

### Experimental details

Tetraphenylcyclopentadienone, Dimethyl Malonate, TiCl4, Pyridine (dry), Dichloromethane, were purchased from commercial sources. The Dichloromethane was dried with molecular sieves. ^1^H and 13C NMR spectra were recorded for solutions in CDCl_3_ on a Bruker Avance III 500 MHz BBFO Probe with the solvent residual proton signal. Flash column chromatography was performed using Silicycle Silica gel 60 (7–230 Mesh, pH 7).

### Synthesis of compound 1a

TiCl_4_ (2.2 mL, 3.8 g, 20 mmol) was added dropwise to an ice-cooled solution of Tetraphenylcyclopentadienone (1.5 g, 3.9 mmol) and Dimethylmalonate (1.98 g, 15 mmol, 0.85 mL) in Dichloromethane (50 mL), under Argon atmosphere. After the addition was complete, dry Pyridine (7.5 mL) was added dropwise over 10 minutes. The reaction mixture was allowed to warm to room temperature and stir under Argon overnight. The solution was cooled at 0 °C and HCl 10% (20 ml) was slowly dropped. The mixture was extracted with Dichloromethane (50 mL × 3) and the organic phase was collected and dried with Na_2_SO_4_. After evaporation of the solvent, the crude was obtained as a red powder. The residue was purified by column chromatography, with Hexane/Ethyl Acetate 10:1 as eluent. The product was isolated as a red powder that was filtered with hexane to remove residual Dimethyl malonate (1.8 g, 3.6 mmol, 92%). ^1^HNMR, (500MHz, CDCl_3_) ppm, δ: 7.21 (m, 10H), 7.09 (m, 2H), 7.02 (m, 4H), 6.73 (m, 4H), 3.07 (s, 6H). ^13^CNMR, (140MHz, CDCl_3_) ppm, δ: 164.7, 150.4, 148.1, 135.0, 133.9, 132.7, 131.5, 130.6, 129.9, 127.7, 127.3, 127.1, 126.8, 52.3. ESI mass [M+Na^+^]^+^ calc. 521.1723, exp. 521.1767, [M+H^+^]^+^ calc. 499.1904, exp. 499.1915.

### Synthesis of compound 1b

TiCL_4_ (2.2 mL, 3.8 g, 20 mmol) was added dropwise to an ice-cooled solution of Tetraphenylcyclopentadienone (1.5 g, 3.9 mmol) and Malononitrile (0.5 g, 7.5 mmol) in Dichloromethane (50 mL), under Argon atmosphere. After the addition was complete, dry Pyridine (7.5 mL) was added dropwise over 10 minutes. The reaction mixture was allowed to warm to room temperature and stirred under Argon overnight. The solution was cooled at 0 °C and HCl 10% (20 ml) was slowly dropped. The mixture was extracted with Dichloromethane (50 mL × 3) and the organic phase was collected and dried with Na_2_SO_4_. After evaporation of the solvent, the crude was obtained as a green powder. The residue was purified by column chromatography, with Hexane/Dichloromethane 7:3 as eluent. The product was isolated as a green-yellow powder. (1.5 g, 3.5 mmol, 44%). ^1^HNMR, (500MHz, CDCl_3_) ppm, δ: 7.36 (m, 10H), 7.16 (t, 2H), 7.07 (t, 4H), 6.87 (d, 4H), ^13^CNMR, (140 MHz, CDCl_3_) ppm, δ: 168.48, 151.1, 145.1, 132.4, 131.7, 131.1, 129.7, 129.0, 128.7, 128.6, 127.5, 111.2. ESI mass [M+Na^+^]^+^calc. 455.1519, exp. 455.1519, [M+H^+^]^+^ calc. 433.1699, exp. 433.1694.

### Photophysical measurements

Absorption and photoluminescence spectra were recorded using a Spectra Max M2 spectrometer. The QY were obtained using Phenothiazine in Acetonitrile as a reference, which Φ is 0.01^35^. The degenerate femtosecond pump-probe measurements were performed to study the ultrafast dynamics in fulvene compounds. The spectrum of 390 nm to 450 nm and a pulse duration of ~8 fs of the light source was used in this study. The spectral and temporal profile in Toluene and DMF for all frequencies and concentrations are shown in the SI. The spikes in the spectral profile appear due to the compression of the light source. An average of 50 scans was measured for each forward and backward scan. Five probe wavelengths (398 nm, 403 nm, 410 nm, 424 nm, 434 nm for compound 1a; 397 nm, 410 nm, 423 nm, 434 nm, 444 nm for compound 1b) were selected which showed better SNR. The backward and forward scans were fitted separately and the mean value of both parameters with an error bar is given in Fig. 8. The kinetics shows negative ΔA which implies ground state photobleaching (PB) or stimulated emission (SE) from the first excited state. The PB occurs when the probe wavelength overlaps with the absorption band and SE occurs when the probe wavelength overlaps with the emission band of the molecule. In both the compounds **1a** and **1b**, the kinetics at shorter wavelengths is mainly contributed from both PB and SE. However, the major contribution for the pump-probe signal ΔA at longer wavelengths is from the SE.

The kinetics at three different concentrations was measured for compound **1a** (0.0065 M, 0.004 M and 0.001M). The spikes near the zero delay are due to the coherent artifacts caused by the interference and nonlinear interaction between pump and probe pulses near the zero delay. The kinetics was fitted bi-exponentially and the longer time constants were plotted at different concentrations. The value of the faster time constant is comparable or less than that of the pulse duration and hence the major contributions from the coherent artifact are expected. The longer time constants could be assigned to the recovery rate of the population in the excited state. In the SI, the amplitude or weight factor of the longer time constant is shown. It is evident from that the amplitude values at shorter wavelengths will have a contribution from both PB and SE. As it goes to longer wavelengths, the contribution of PB reduces and enhances the amplitude.

The kinetics at three different concentrations of compound **1b** (0.0032 M, 0.0065 M, 0.002 M) were also performed. However, the sample with the highest concentration was unable to observe any pump-probe signal as the quenching of emission occurred at this concentration. The lowest concentrations show the negative pump-probe signal ΔA and the kinetics have been fitted bi-exponentially.

### Computational details

The density functional theory (DFT) calculations were performed for geometry optimization and eigenvalues of molecular orbitals. The B3LYP exchange-correlation functional^68–70^ and the double numerical basis set with polarization (DNP) implemented in the DMol^3^ program package^71,72^ were employed for all numerical calculations.

### Supporting Informations

The Supporting Information provides all experimental data that are cited and not showed in the paper like the UV spectra in DMF, Toluene, Methanol, CH_2_Cl_2_, the PL spectra of compounds **1a**, **1b** and **3** in Toluene, CH_3_CN, Methanol, CH_2_Cl_2_, all femtosecond spectroscopy experiments, all UV visible and PL spectra in the mixtures MeOH/Glycerol.

## Supplementary Information

Supplementary information
