# Supplementary material for: Tuning of the Electro-Optical Properties of Tetraphenylcyclopentadienone via Substitution of Oxygen with Sterically-Hindered Electron Withdrawing Groups
Source: Sci Rep. 2019 Sep 4;9:12762. doi: 10.1038/s41598-019-49303-w (PMC6726659; doi:10.1038/s41598-019-49303-w)
Supplement: Supplementary file 1 — Supplementary information [file 41598_2019_49303_MOESM1_ESM.docx]

**Supporting information**

**Tuning of the Electro-Optical Properties of Tetraphenylcyclopentadienone via Substitution of Oxygen with Sterically-Hindered Electron Withdrawing Groups.**

Carmine Coluccini^a^, Puliparambil Thilakan Anusha^b,c^, Hsin-Yi Tiffany Chen^d^, Sheng-Lun Liao^e^, Ying Kuan Ko^b^, Atsushi Yabushita^b^, Chih Wei Luo^b,f^, Yoke Mooi Ng^a^ and Yit Lung Khung^g^[[1]](#footnote-1)^*^

*^a^ Institute of New Drug Development, China Medical University, No.91 Hsueh-Shih Road, Taichung 40402, Taiwan*

*^b^ Department of Electrophysics, National Chiao Tung University, 1001 University Road, Hsinchu 30010, Taiwan*

*^c^Department of Physics, School of Advanced Sciences, Vellore Institute of Technology, Vellore 632 014, India*

*^d^ Department of Engineering and System Science, National Tsing Hua University, Hsinchu 30013, Taiwan.*

*^e^ Molecular Science Center, GGA Corp., Taipei 11493, Taiwan*

*^f^ Taiwan Consortium of Emergent Crystalline Materials (TCECM), Ministry of Science and Technology, Taiwan*

*^g^ Department of Biological Science and Technology, China Medical University, No.91 Hsueh-Shih Road, Taichung 40402, Taiwan*

The Suppporting Informations provide all files of the experimental part of the work. The figures S1-S4 concern all the NMR characterization, 1 H and 13 C spectra of the synthesized compounds. The figure S5 display all UV-visible spectra of compounds in different solvents (Toluene, CH_2_Cl_2_, MeOH, CH_3_CN, DMF). In the figure S6 are reported all PL spectra in different solvents (Toluene, CH_2_Cl_2_, MeOH, CH_3_CN) of compounds **1a** and **1b**. Each graphic contains the emission spectra at two different concentrations. In the figure S7 are reported the emission spectra of compound **3** in the same solvents. In the figures S8-S14 are reported all graphic of the Time resolved laser spectroscopy. The figure S15 display the emission of compound **1b** at concentration of 23 × 10^-7^ M. The figure S16 shows the calculated optimized geometries of compounds **1a**, **1b**, **3**.

The quantum yields of the table 1 were calculated according with the following formula:

Φ_s_ = (n_s_/n_r_)*( I_s_/I_r_)*[(1-10^-Ar^)/(1-10^-As^)]* Φ_r_

Φ_s_=Quantum yield of the sample

Φ_r_=Quantum yield of the reference

n_s_= refraction index of the solvent of the sample

n_r_=refraction index of the solvent of the refery

I_s_= emission intensity of the sample (area under the curve of emission)

I_r_= emission intensity of the refery (area under the curve of emission)

A_s_=Absolute absorbance of the sample at the concentration of emission

A_s_=Absolute absorbance of the refery at the concentration of emission.


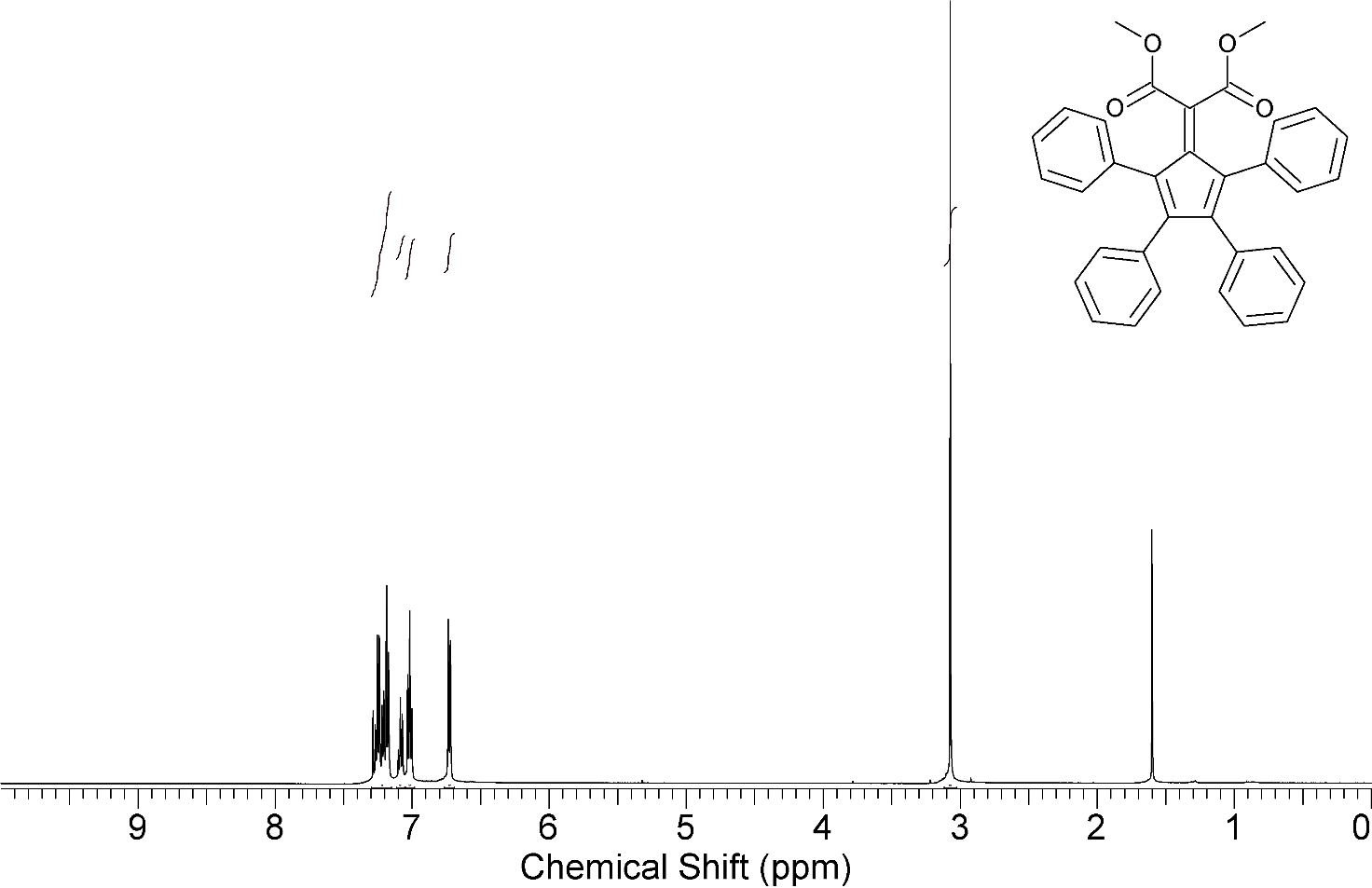
**Figure S1.** ^1^HNMR of compound **1a**.


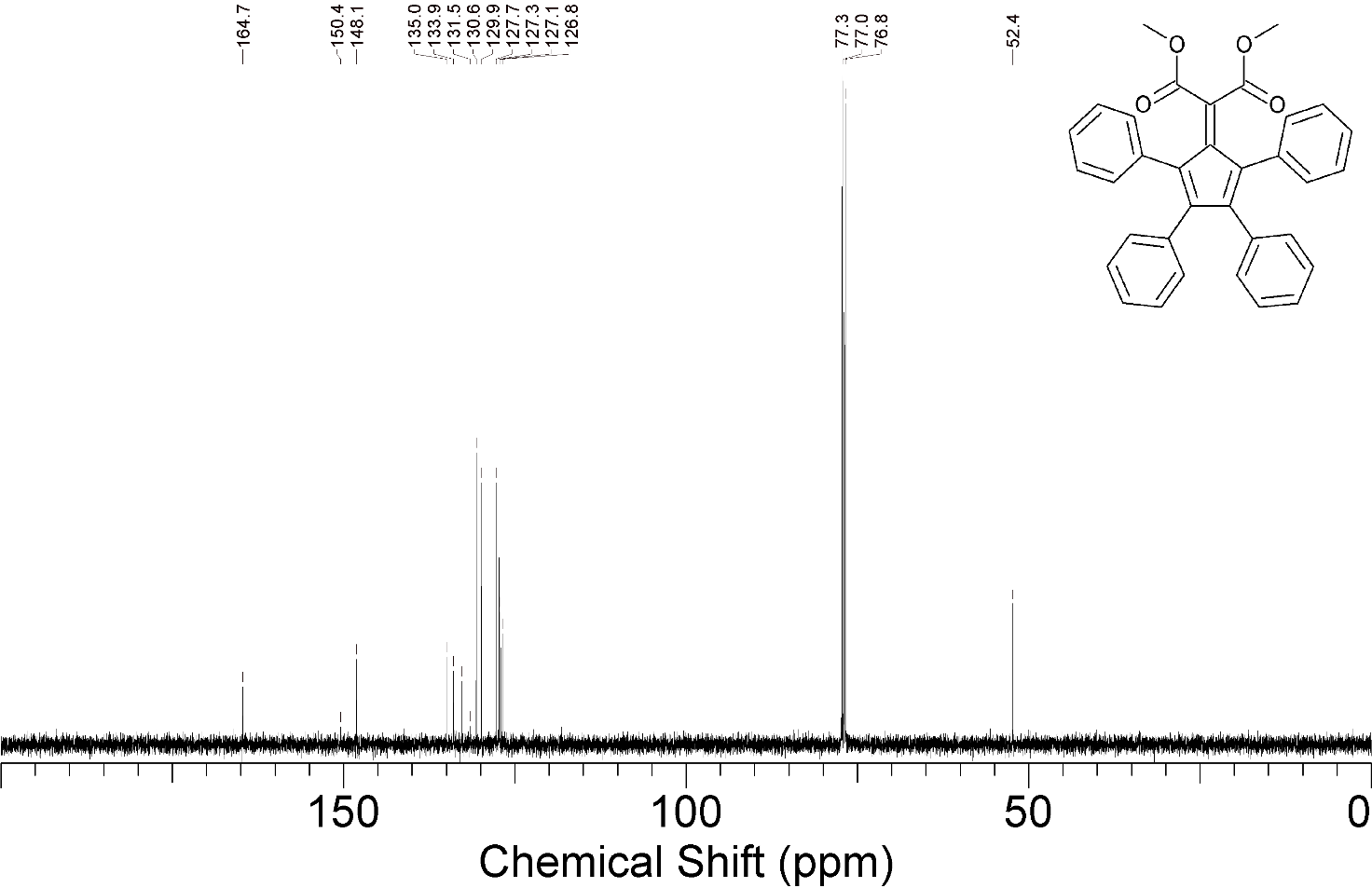


**Figure S2.** ^13^CNMR of compound **1a**.


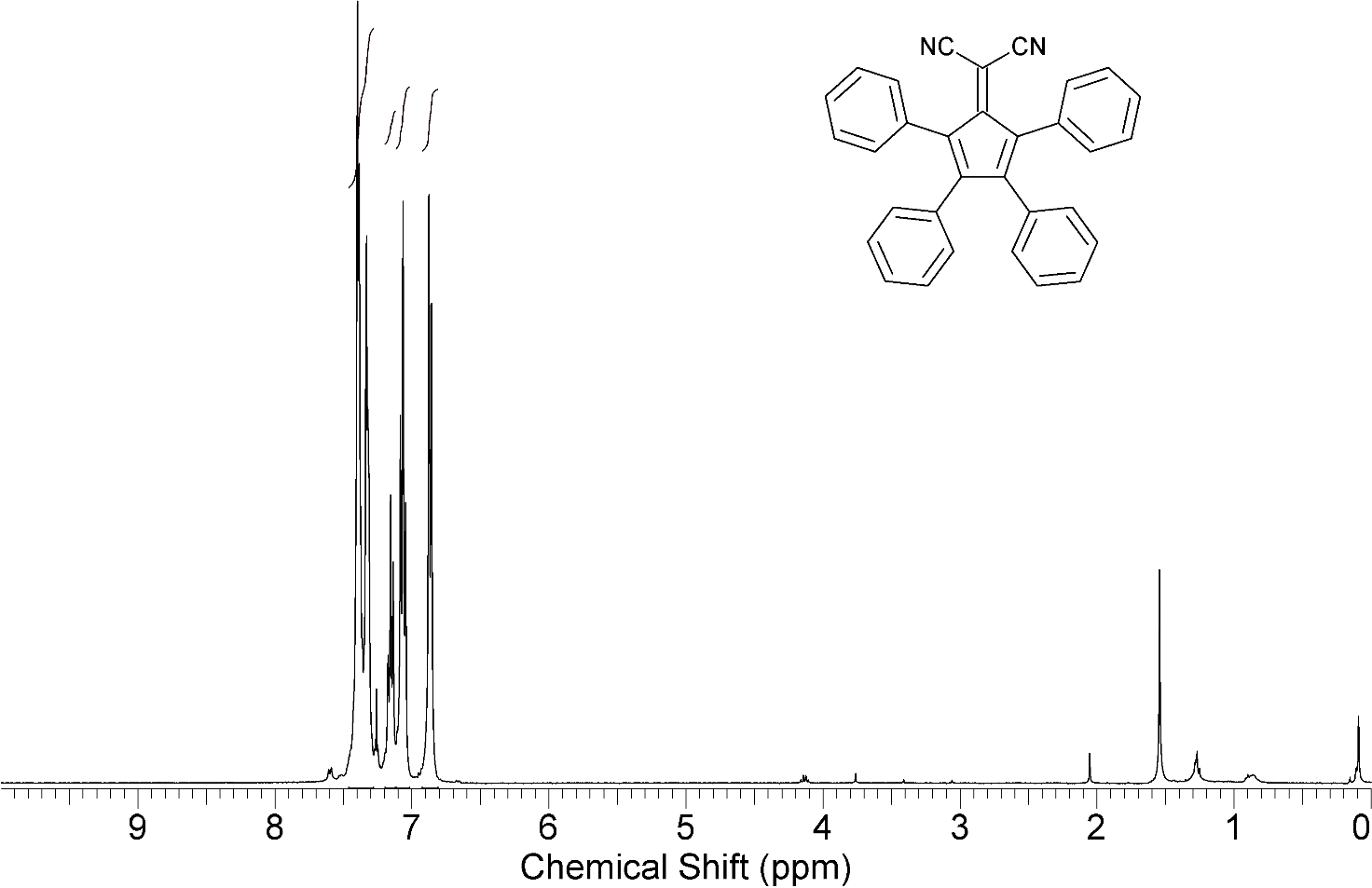


**Figure S3.** ^1^HNMR of compound **1b**.


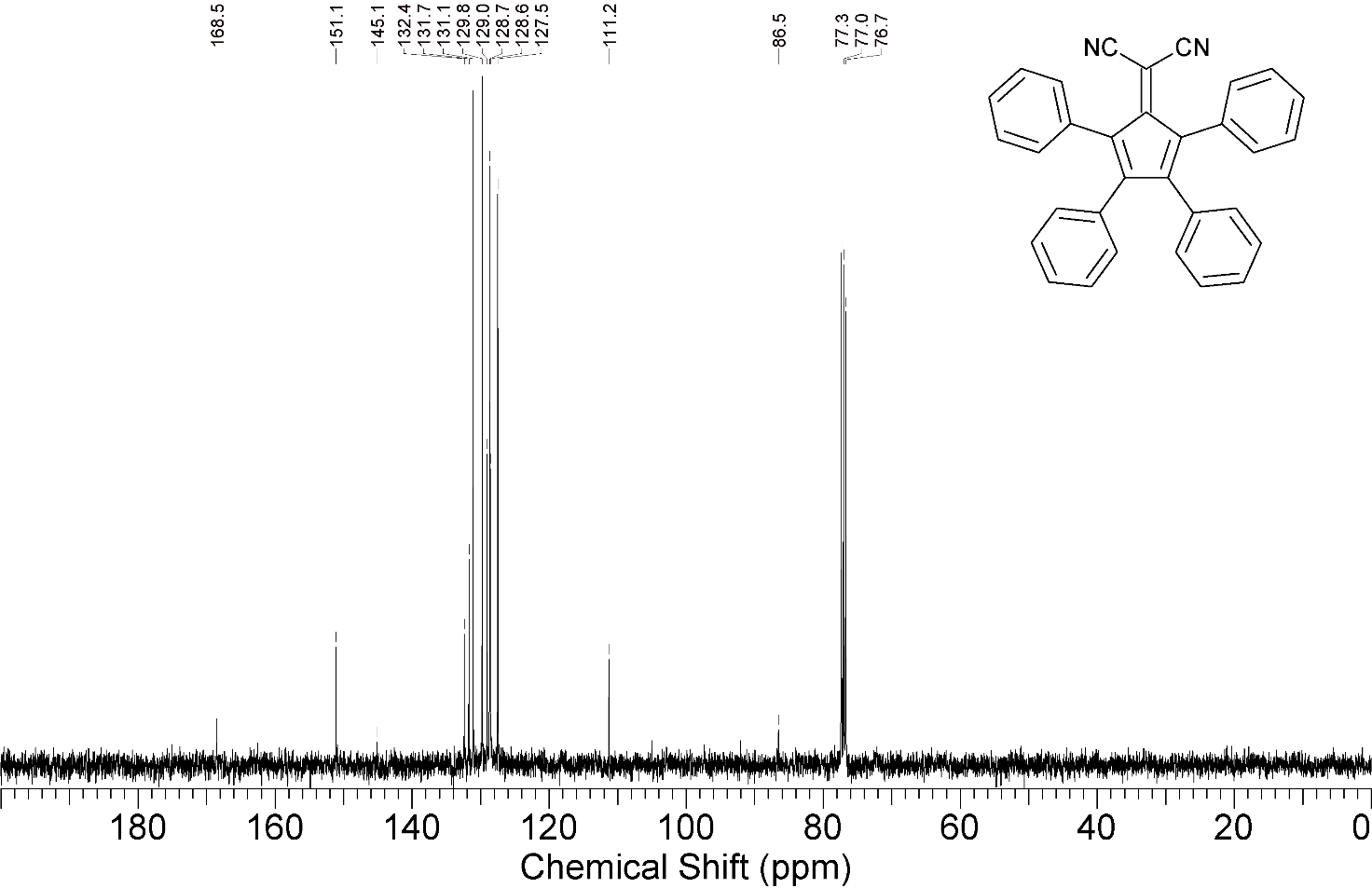


**Figure S4.** ^13^CNMR of compound **1b**.

**
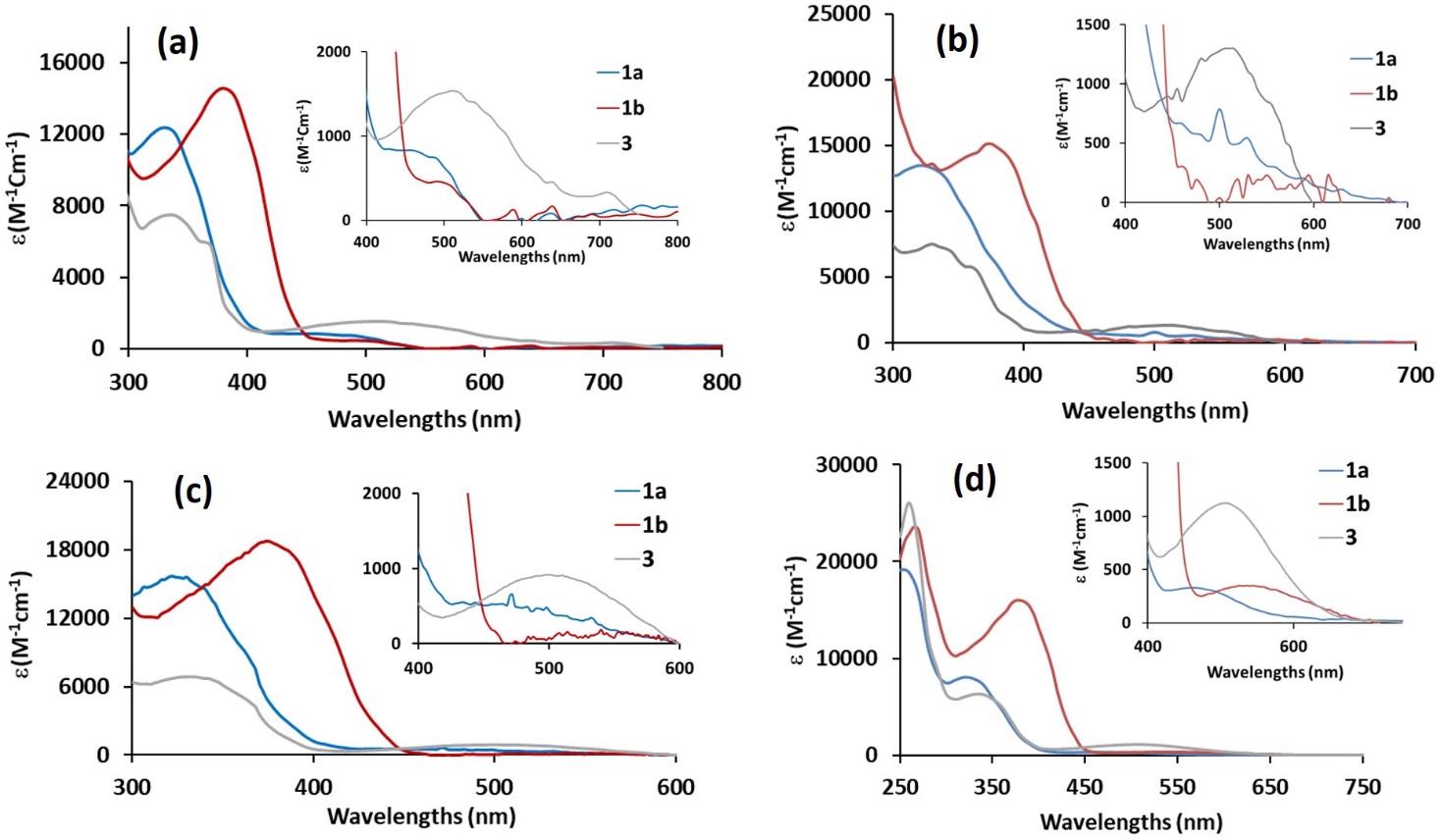
**

**Figure S5.** UV-visible spectra in Toluene (a), DMF (b), Methanol (c), CH_2_Cl_2_ (d). The insets are the spectra in the range 400-800 nm.


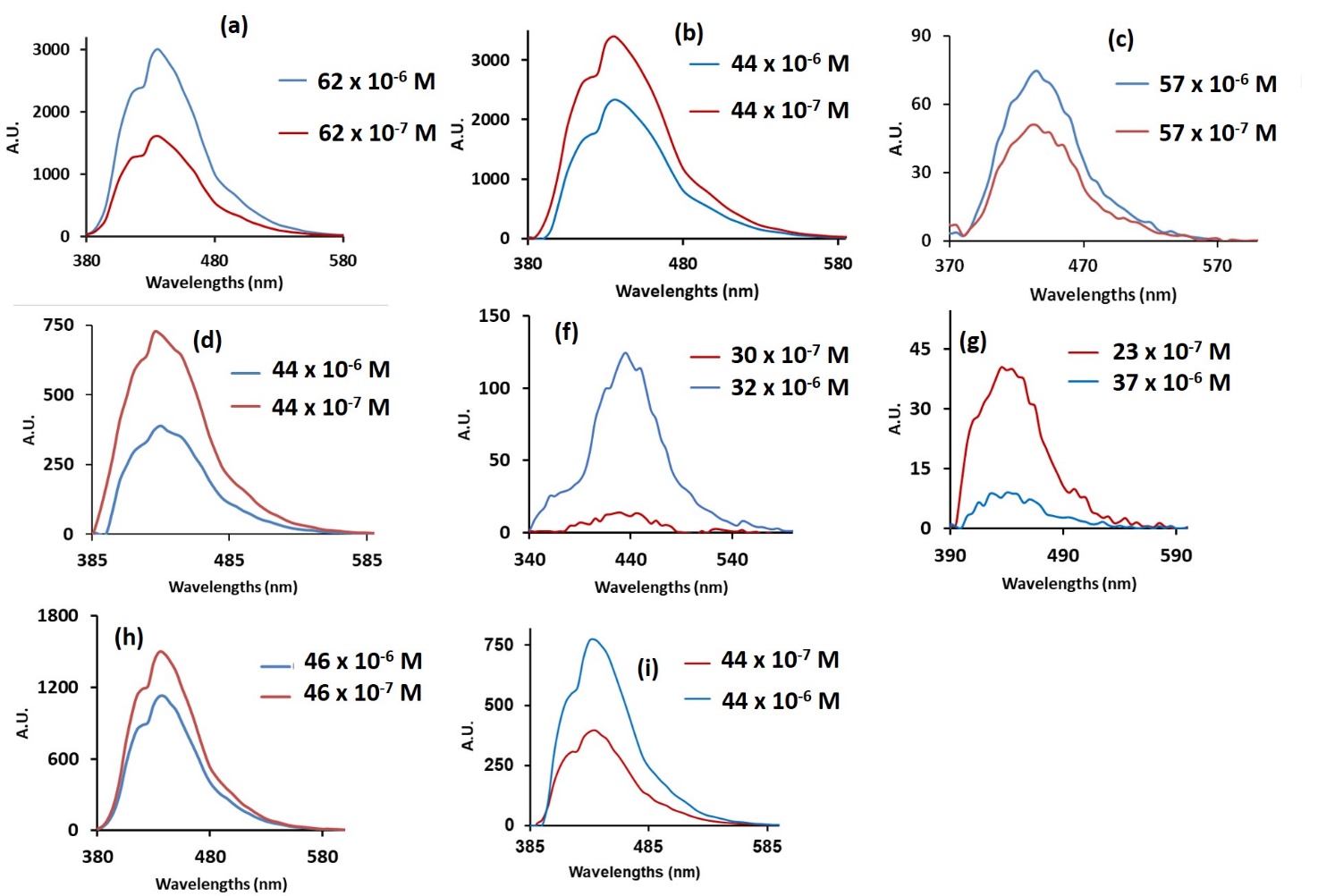


**Figure S6.** PL spectra of compound **1a** in Toluene (a), **1b** in Toluene (b), **1a** in CH_3_CN (c), **1b** in CH_3_CN (d), **1a** in MeOH (f), **1b** in MeOH (g), **1a** in CH_2_Cl_2_ (h), **1b** in CH_2_Cl_2_ (i).


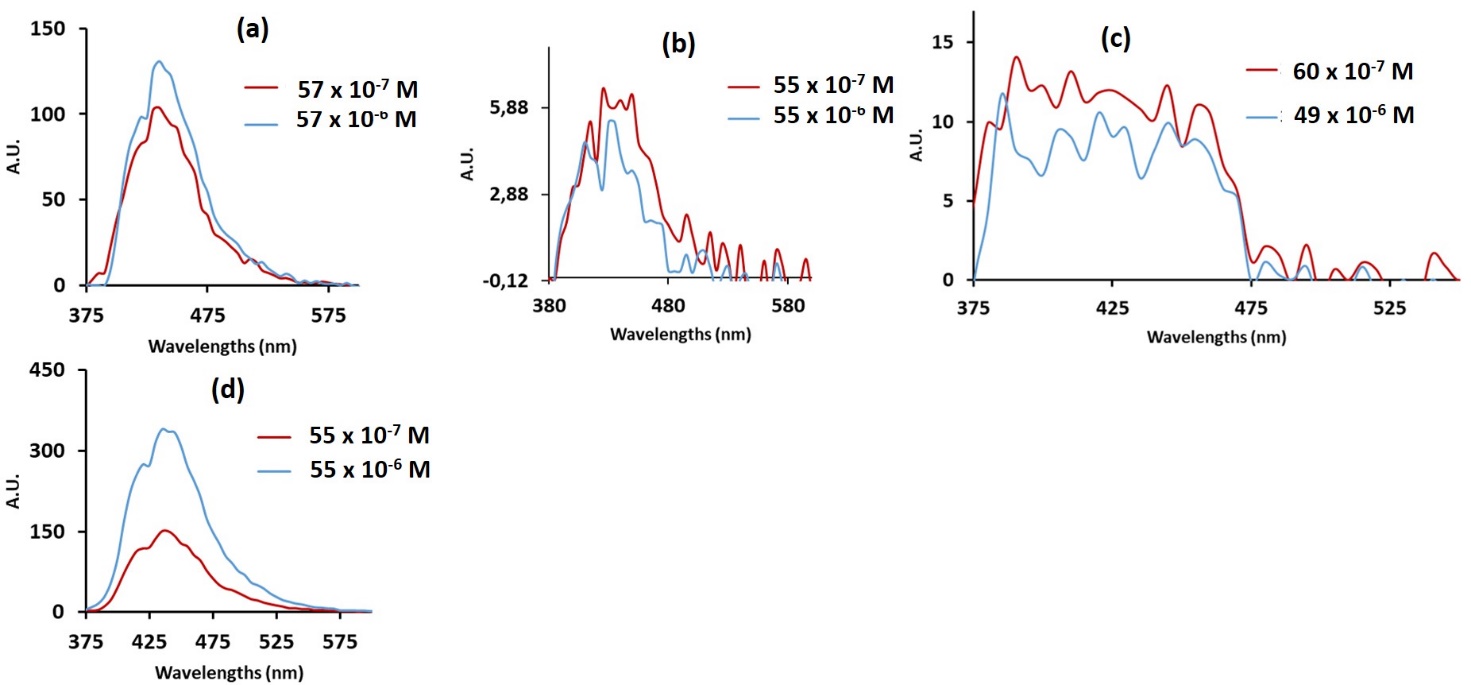


**Figure S7.** PL spectra of compound **3** in Toluene (a), in CH_3_CN (b), in MeOH (c), in CH_2_Cl_2_ (d).

**
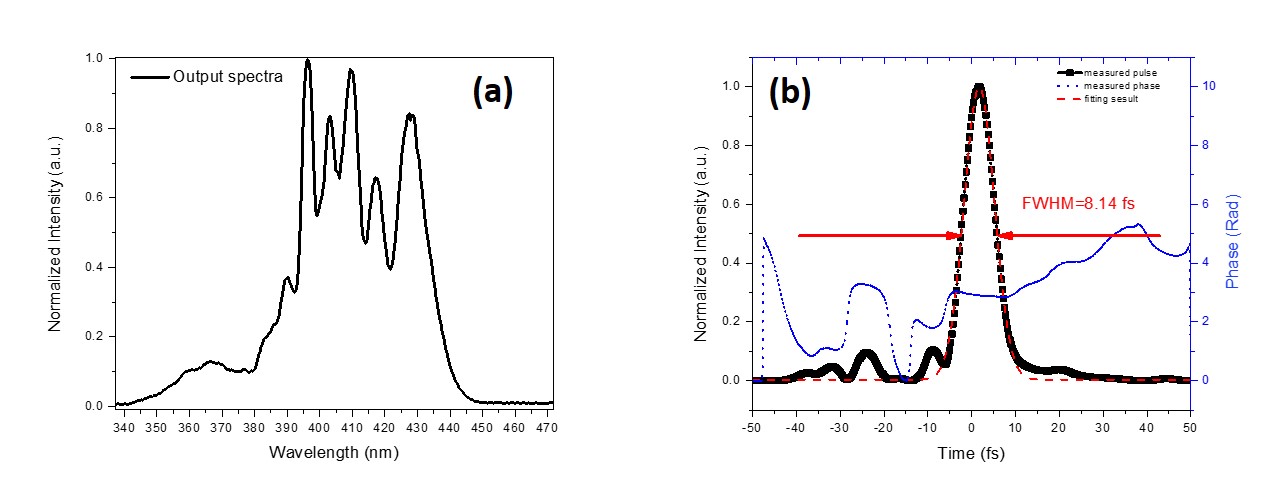
**

**Figure S8.** Spectra profile (a) and temporal profile (b) of 400 nm laser pulse. Dotted blue line shows the measured phase of the femtosecond pulse by FROG measurements.


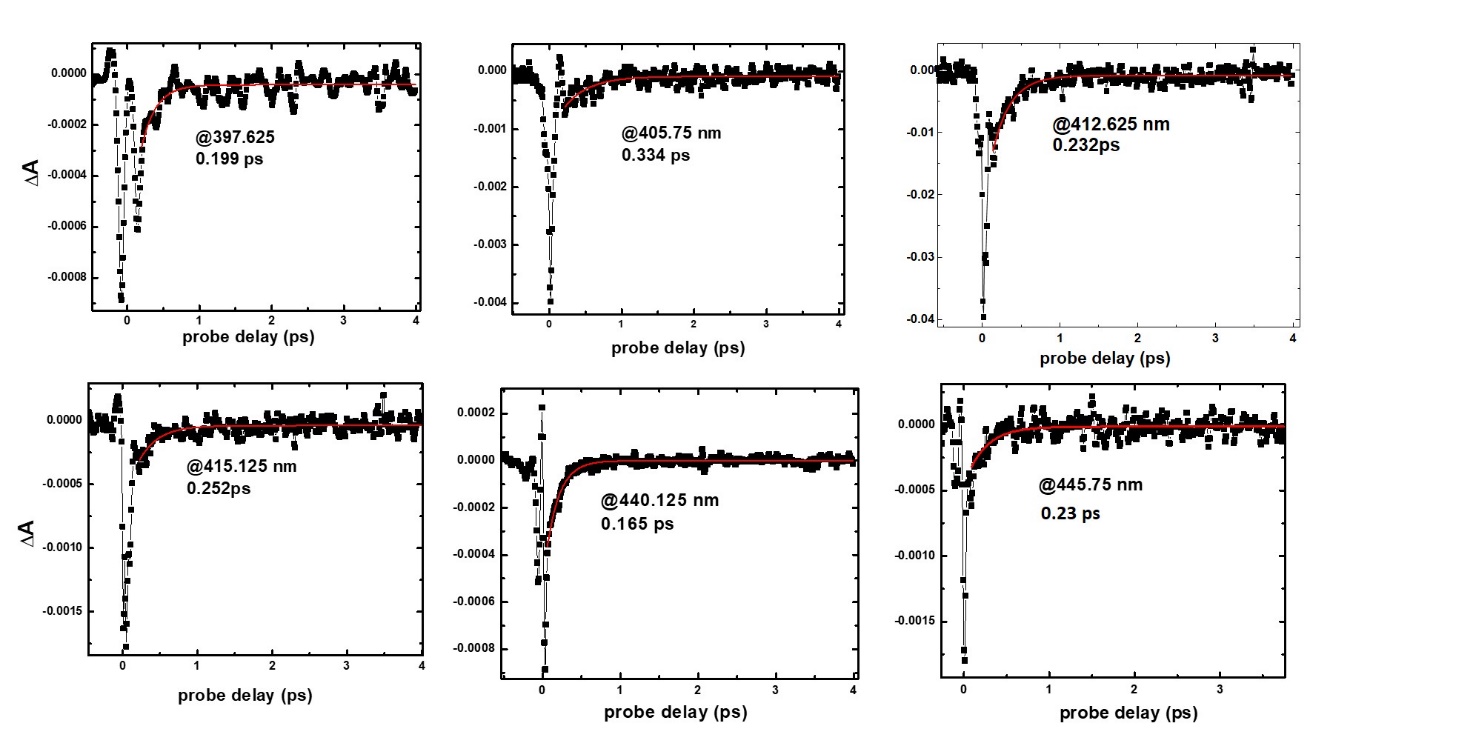


**Figure S9.** Temporal dynamic at various wavelengths for compound **1a** in DMF

**
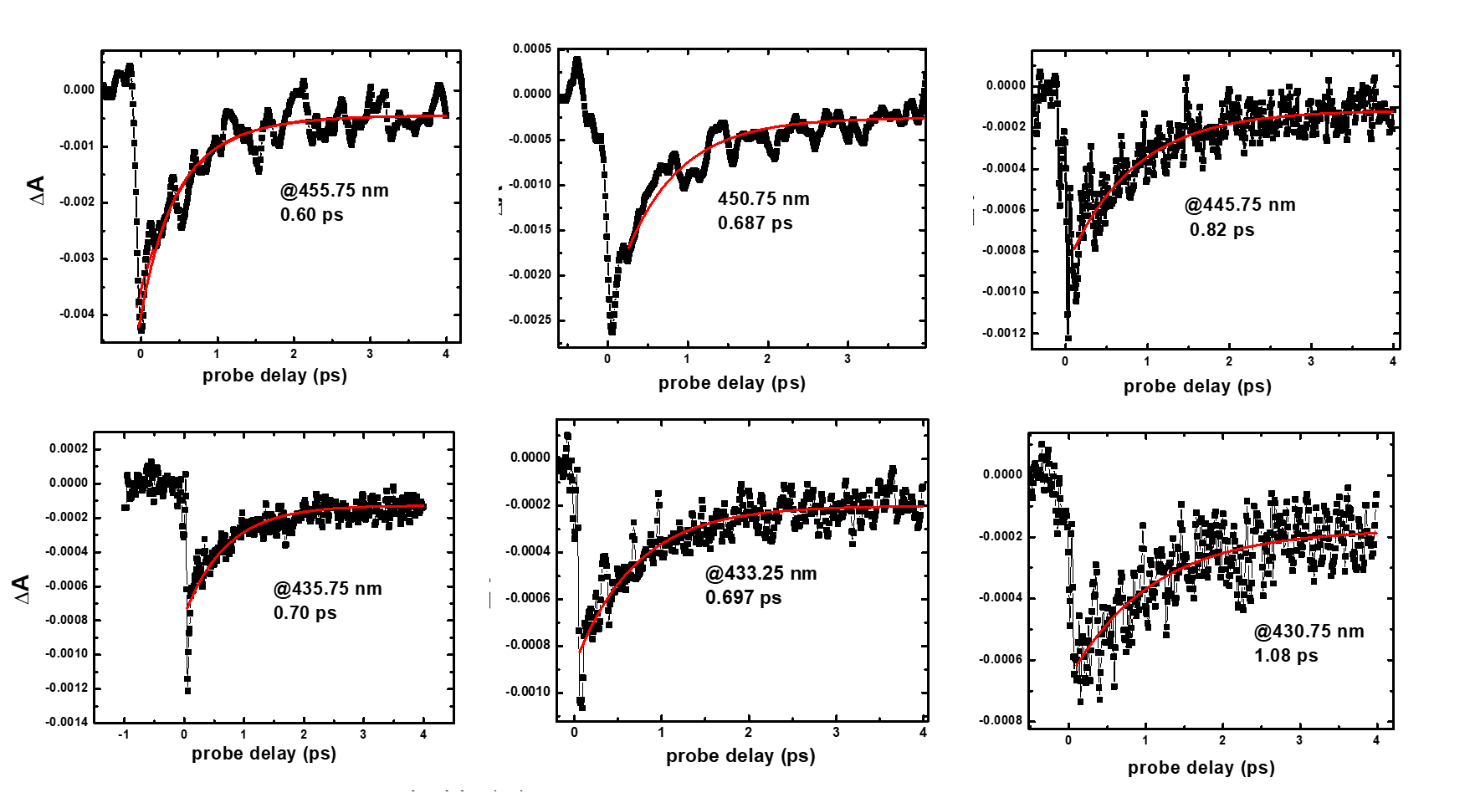
**

**Figure S10.** Temporal dynamic at various wavelengths for compound **1b** in DMF


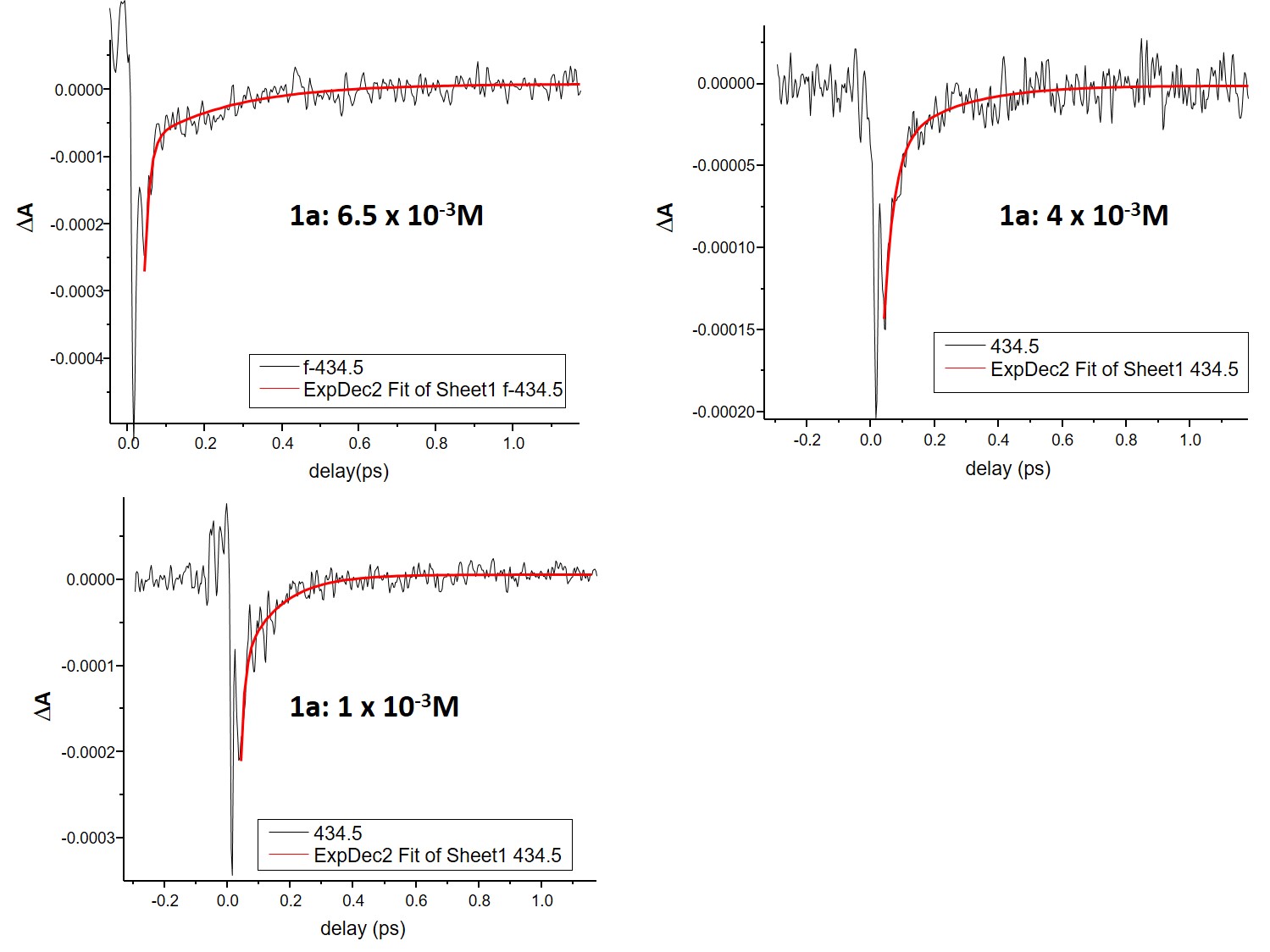


**Figure S11.** Kinetic traces of compound 1a at different concentrations in toluene at 434.5 nm probe wavelength.

**
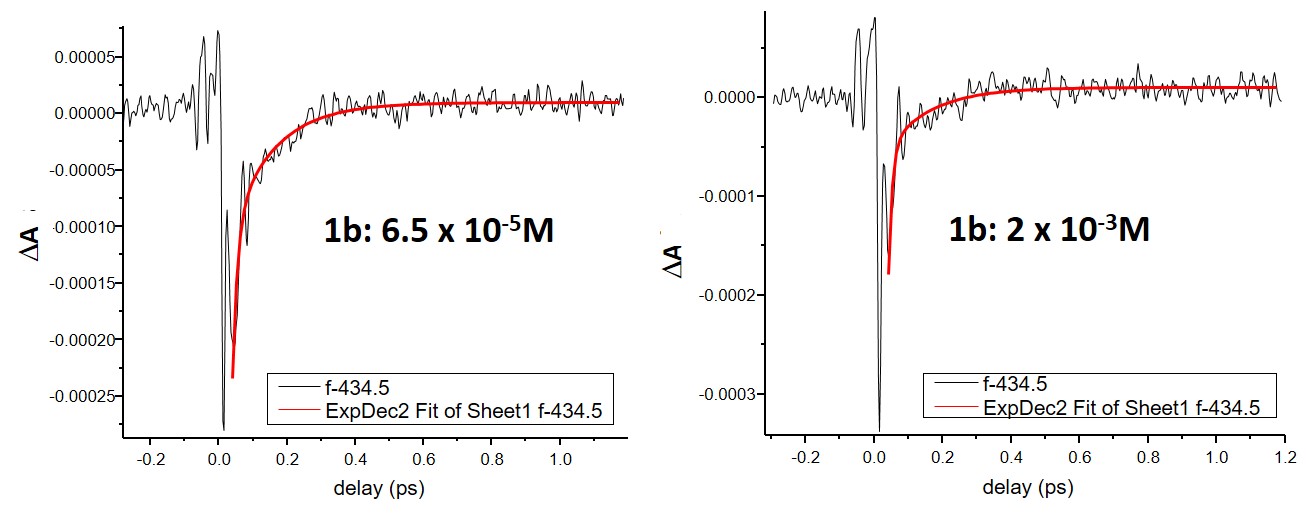
**

**Figure S12.** Kinetic traces of compound 1b at different concentrations in Toluene at 434.5 nm probe wavelength.

**
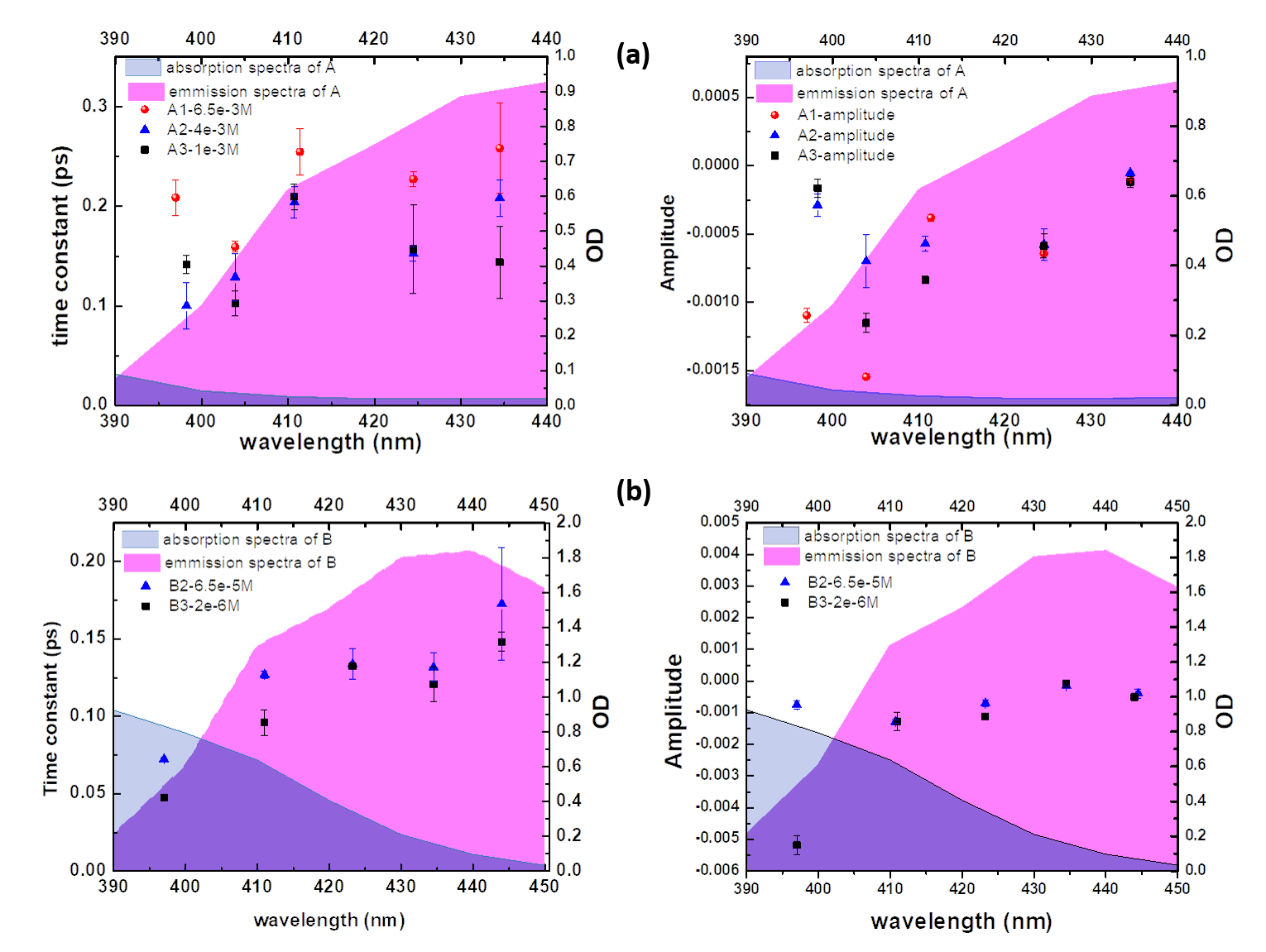
**

**Figure S13.** Fitted parameters (bi-exponential) for compound **1a** (a) and **1b** (b) in Toluene.


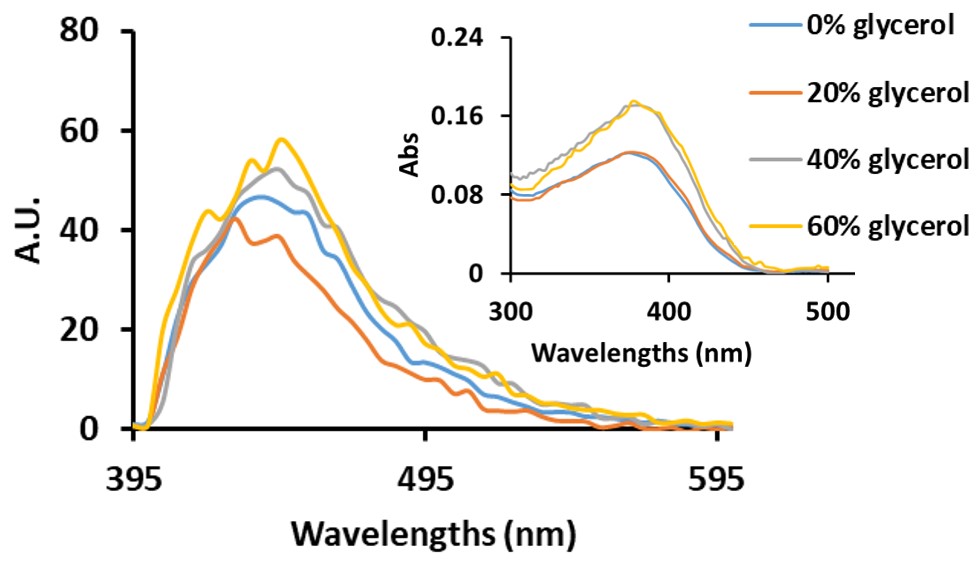


**Figure S15.** PL spectra and UV-vis spectra (inset) of compound **1b** 23 × 10^-7^ M in mixtures MeOH/Glycerol. The enhancement of the percentage of Glycerol and the consequent increased viscosity does not provide a sensible change of the UV spectrum and a growing of the emission.


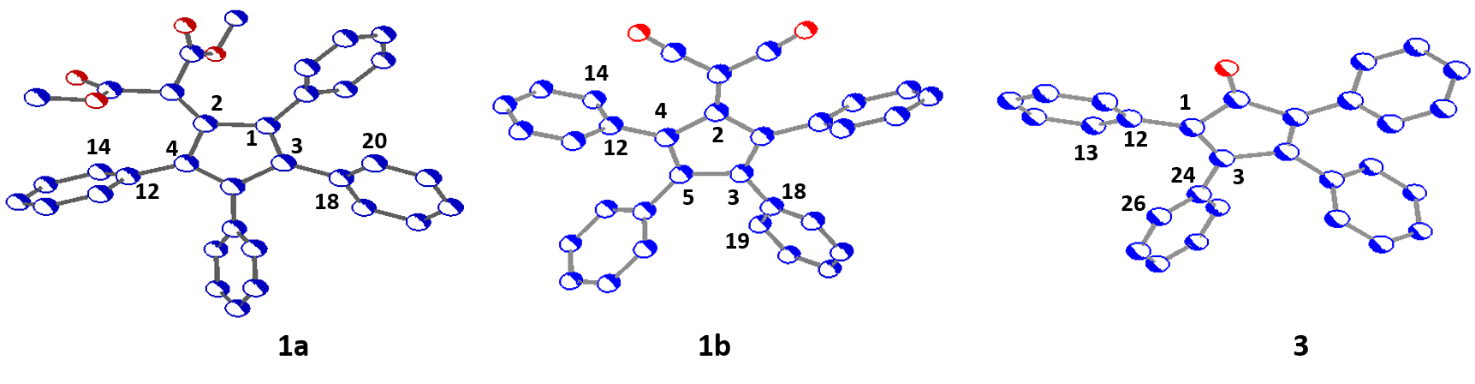


**Figure S16.** Optimized geometries of compounds **1a**, **1b**, and **3**. The dihedral angles C2-C4-C12-C14 and C1-C3-C18-C20 of compound **1a** are 63.01° and 52.54° respectively. The dihedral angles C2-C4-C12-C14 and C5-C3-C18-C19 of compound **1b** are 80.88° and 43.42° respectively. The dihedral angles C3-C1-C12-C13 and C1-C3-C24-C26 of compound **3** are 39.28° and 50.17° respectively.

1. ^*^Dr. Yit Lung Khung (Corresponding author). Tel.: +886 04-22022205; e-mail: yitlung.khung@mail.cmu.edu.tw [↑](#footnote-ref-1)
